# Iron single atom enzyme-mediated hydrogen sulfide delivery amplifies reactive oxygen species cascade to induce ferroptosis susceptibility

**DOI:** 10.1016/j.mtbio.2025.102184

**Published:** 2025-08-09

**Authors:** Xuegang Niu, Penghui Wei, Mingtao Zhu, Hongjia Zheng, Dezhi Kang, Qianxi Chen, Fuxiang Chen, Yiping Li, Rong Xie, Yang Zhu, Dengliang Wang

**Affiliations:** a Department of Neurosurgery, Neurosurgery Research Institute, The First Affiliated Hospital, Fujian Medical University, Fuzhou, Fujian, 350005, China; b Department of Neurosurgery, National Regional Medical Center, Binhai Campus of the First Affiliated Hospital, Fujian Medical University, Fuzhou, 350212, Fujian, China; c Department of Neurosurgery, Xuanwu Hospital, Capital Medical University, Beijing, 100053, China; d Department of Neurosurgery, Xiamen Xinglin Hospital (Xiamen Infectious Disease Hospital), Xiamen, 361000, China; e Department of Neurosurgery, Huashan Hospital, Fudan University, Shanghai, 200040, China; f Shaowu Municiple Hospital of Fujian Province, China; g Fujian Provincial Institutes of Brain Disorders and Brain Sciences, The First Affiliated Hospital, Fujian Medical University, Fuzhou, 350005, China

**Keywords:** Single atom enzyme, Hydrogen sulfide, Ferroptosis, Catalytic therapy, Reactive oxygen species

## Abstract

Lipid peroxidation (LPO) represents one of the most deleterious processes contributing to ferroptosis susceptibility. However, the tumor microenvironment is often characterized by an overproduction of endogenous glutathione (GSH) and reactive oxygen species (ROS)-scavenging enzymes, which limit the ferroptosis susceptibility. Herein, we introduce an iron single-atom enzyme (Fe/SAE) nanoplatform, termed Fe/SAE@A, that acts as hydrogen sulfide (H_2_S) donor anethole trithione (ADT) delivery system to amplify LPO-mediated ferroptosis vulnerability. Upon internalization by cancer cells, Fe/SAE, featuring atomically dispersed active metal sites, exhibits remarkable peroxidase-mimicking activity, converting hydrogen peroxide (H_2_O_2_) into hydroxyl radicals. Furthermore, Fe/SAE@A facilitates the release of ADT, delivering H_2_S to significantly inhibit ROS-scavenging enzymes, which results in elevated intracellular H_2_O_2_ levels. This, in turn, initiates a robust Fe/SAE-catalyzed ROS cascade within cancer cells, driving irreversible LPO. Additionally, Fe/SAE@A exhibits glutathione oxidase-mimicking activity, efficiently oxidizing reductive GSH to glutathione disulfide, thereby promoting the inactivation of glutathione peroxidase 4. These results confirm the mechanistic basis of ferroptosis induced by Fe/SAE@A, underscoring remarkable capacity to deliver H_2_S, ignite ROS storm, and consumes GSH, all of which enhance ferroptosis susceptibility. This work offers critical insights into leveraging H_2_S-releasing cascade SAE for potentiating ferroptosis vulnerability in cancer cells.

## Graphical abstract

An iron single-atom enzyme (Fe/SAE) nanoplatform, referred to as Fe/SAE@A, serves as a hydrogen sulfide (H_2_S) donor and anethole trithione (ADT) delivery system. Fe/SAE@A releases H_2_S, which significantly inhibits reactive oxygen species (ROS)-scavenging enzymes, such as thioredoxin reductase (TrxR) and catalase (CAT), leading to elevated intracellular hydrogen peroxide (H_2_O_2_) levels. Moreover, Fe/SAE@A exhibits excellent peroxidase (POD) and glutathione oxidase (GSHOX)-mimicking activities, effectively converting H_2_O_2_ into hydroxyl radicals (•OH) and oxidizing glutathione (GSH) to glutathione disulfide (GSSG), thereby enhancing vulnerability to ferroptosis.

**Figure:**
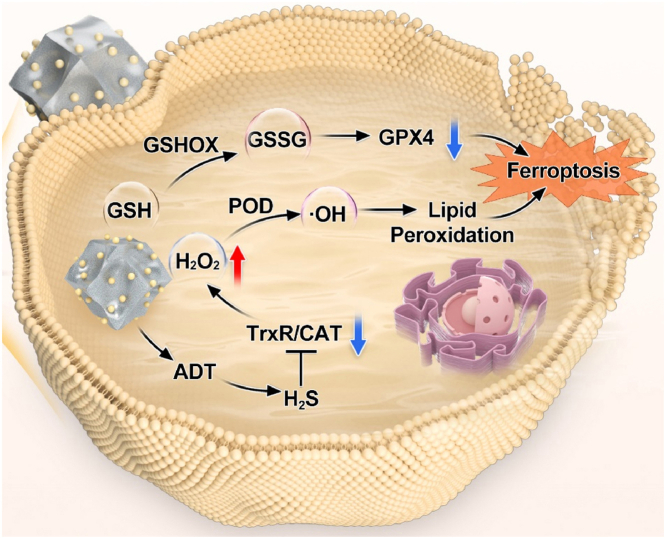

## 1 Introduction

Ferroptosis is a unique and regulated form of cell death that is dependent on iron, characterized by the iron-catalyzed accumulation of lethal lipid peroxidation (LPO) and alterations of mitochondrial morphology, which ultimately results in plasma membrane destruction [[1], [2], [3], [4], [5]]. Tumor cells, owing to their increased iron demands and elevated reactive oxygen species (ROS) production during rapid proliferation, demonstrate an enhanced vulnerability to ferroptosis [[6], [7], [8], [9], [10]]. Furthermore, develop an increasing reliance on lipid hydroperoxidase-glutathione peroxidase 4 (GPX4) for survival, rendering them more susceptible to ferroptosis [[11], [12], [13], [14]]. Glutathione (GSH), an endogenous reductive molecule, acts as a substrate for GPX4, facilitating the conversion of lethal LPO into non-toxic derivatives, thereby suppressing ferroptosis [[15], [16], [17], [18]]. Hydrogen sulfide (H_2_S), an endogenous gas signaling molecule, regulates a range of physiological processes, including cell proliferation, anti-inflammatory responses, and GSH synthesis, positioning it as a natural regulator of intracellular redox homeostasis [[19], [20], [21], [22]]. In addition, the intricate endogenous redox balance is tightly regulated not only by GSH but also by elevated content of ROS-scavenging enzymes present in the tumor microenvironment (TME), a phenomenon that has been frequently observed across numerous types of malignant cancers. Fortunately, the elevated levels of H_2_S can suppress the reactivity of endogenous enzymes, further enhancing ROS levels [[23], [24], [25]]. Therefore, it is crucial to delivery H_2_S as a therapeutic target for the modulation of ferroptosis.

Nanozymes, representing a novel category of nanomaterials, possess catalytic properties that closely resemble those of natural enzymes. This remarkable similarity has not only garnered significant attention but also paved the way for innovative therapeutic strategies, particularly in the realm of treating diseases that are mediated by ROS [[26], [27], [28], [29], [30]]. Nanozymes possess excellent physical and chemical properties, along with significant catalytic activity, making them widely biomedical applications [[31], [32], [33], [34]]. However, challenges such as the diversity of nanostructures, low substrate selectivity, and unacceptable atomic utilization efficacy continue to limit their further development and application [[35], [36], [37], [38]]. Lately, cutting-edge single atom nanozymes, characterized by maximum atom utilization efficiency, extremely high catalytic activity, and clear coordination environment, making them widely applicable in both in vitro detection and in vivo treatment [[39], [40], [41], [42], [43]]. Due to their ability to efficiently generate sufficient ROS, peroxidase (POD)-like SAEs, which catalyze hydrogen peroxide (H_2_O_2_) into hydroxyl radicals (•OH), have garnered considerable interest in ferroptosis [[44], [45], [46], [47]]. However, the abundance of endogenous ROS-scavenging enzymes and reductive GSH in TME still inhibits the sustained accumulation of LPO, thereby hindering the therapeutic efficacy of SAE-induced ferroptosis [[48], [49], [50], [51], [52]]. Thus, strategies to inhibit reductases and deplete GSH in the TME to promote the ROS cascade of SAE are of critical importance for inducing irreversible ferroptosis.

**Scheme 1:**
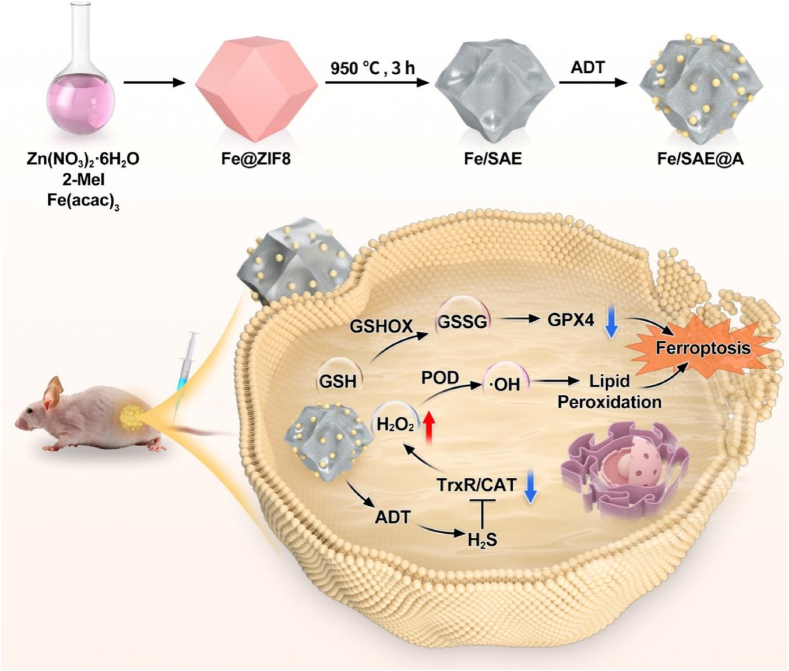
Schematic Illustration for Anticancer Treatment of Fe/SAE@A.

Encouraged by the aforementioned concept, a proof-of-concept iron (Fe) SAE with Fe-N_4_ active sites, loaded with Food and Drug Administration (FDA)-approved H_2_S donor anethole trithione (ADT), is developed (Fe/SAE@A) to enhance ferroptosis susceptibility (Scheme 1). In the Fe/SAE structure, the nitrogen-doped carbon (N-C) nanoframework serves as a robust scaffold to immobilize isolated Fe^δ+^ ions (where 0 <δ < 3), each coordinated with four nitrogen atoms. This strategic design not only ensures the stability of the active sites but also maximizes their exposure. The expanded spacing of the graphite layers further facilitates the accessibility of these active sites, thereby significantly enhancing the catalytic activities of the material. Fe/SAE@A exhibits POD-like catalytic activities, effectively converting H_2_O_2_ into •OH in the TME, thereby inducing lethal LPO. Moreover, Fe/SAE@A produces H_2_S to inhibit the activity of ROS-scavenging enzymes (TrxR and CAT) and elevates the H_2_O_2_ level, which in turn amplified Fe/SAE@A-mediated POD-like efficacy. Importantly, Fe/SAE@A also demonstrates glutathione oxidase (GSHOX)-mimicking activity, efficiently oxidizing reductive GSH to glutathione disulfide (GSSG), thereby inactivating GPX4. This dual-action mechanism creates a powerful effect: while ROS directly attack tumor cells, H_2_S suppresses the tumor's defense mechanisms, thereby enhancing the overall therapeutic efficacy. These experiments demonstrate that Fe/SAE@A efficiently induces irreversible tumor ferroptosis via the accumulation of LPO, inhibition of ROS-scavenging enzymes, and inactivation of GPX4. This study highlights the potential of H_2_S-amplified ROS cascade tactics for SAE-mediated TME regulation to strengthen the efficacy of ferroptosis.

## 2 Result and discussion

### 2.1 Synthesis and Characterization of Fe/SAE

**Fig. 1:**
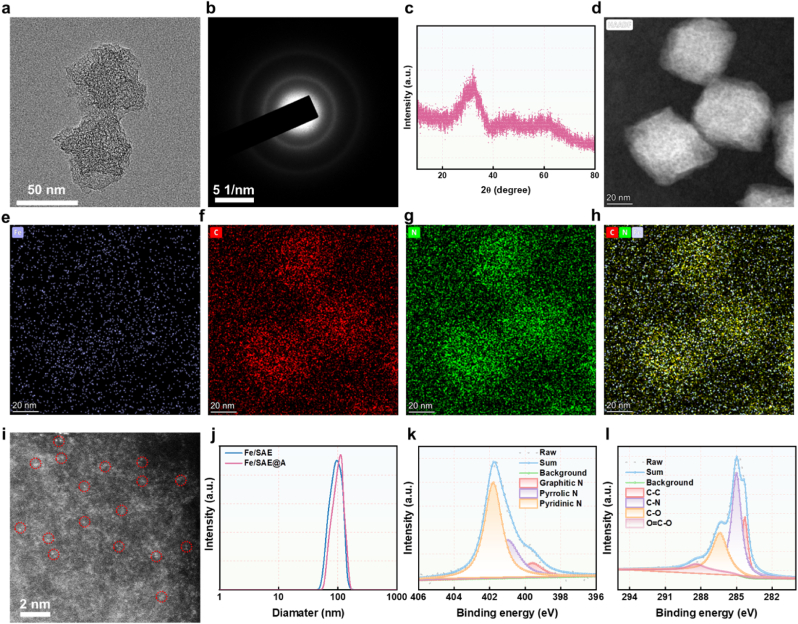
**Characterization of Fe/SAE.** (a) TME image of Fe/SAE. (b) SAED image of Fe/SAE. (c) XRD pattern of Fe/SAE. (d–h) EDX mapping image of Fe/SAE. (i) HAADF-STEM image of Fe/SAE. (j) DLS of Fe/SAE and Fe/SAE@A. (k) High-resolution XPS C 1s and (l) N 1s spectra in Fe/SAE.

The intricate process of synthesizing Fe/SAE is meticulously depicted in Scheme 1. First, Fe/SAE was fabricated via pyrolysis of Fe@ZIF-8 precursor. Subsequently, iron acetylacetonate (Fe(acac)_3_) was encapsulated in situ within the ZIF-8 host cage, effectively integrating the iron precursor into the metal-organic framework. Transmission electron microscopy (TEM) images revealed that the Fe@ZIF-8 precursors retained a rhombic dodecahedron morphology (Fig. S1). After annealing the Fe@ZIF-8 derivatives, Fe/SAE was successfully obtained. High-resolution TEM images revealed that Fe/SAE exhibited a uniform dodecahedron shape with a diameter of about 60 nm. The surface of Fe/SAE became rough and porous after 950° treatment (Fig. 1a). Selected area electron diffraction (SAED) images of Fe/SAE displayed two diffuse diffraction rings, indicative of amorphous N-C nanoframeworks (Fig. 1b). X-ray diffraction (XRD) pattern showed no crystalline peaks for metallic Fe or Fe oxide, suggesting no significant Fe aggregation and supporting the successful creation of a single-atomic Fe nanozyme (Fig. 1c). Energy-dispersive X-ray spectroscopy (EDS) mapping showed a uniform distribution of Fe, C, and N atoms within the Fe/SAE (Fig. 1d–h). Aberration-corrected atomic-resolution high-angle annular dark-field scanning transmission electron microscopy (HAADF-STEM) displayed bright dots, highlighted by red circles, representing atomically distributed Fe (Fig. 1i). The Fe loading efficiency in Fe/SAE was determined to be 0.82, as measured by inductively coupled plasma mass spectrometry (ICP-MS). The ADT was incorporated into Fe/SAE via hydrophobic interactions to prepare Fe/SAE@A. The hydrodynamic size of both Fe/SAE and Fe/SAE@A was about 100 nm, as measured by dynamic light scattering (DLS) (Fig. 1j). And zeta potential of both Fe/SAE and Fe/SAE@A were found to be approaching neutral values (Fig. S2). In addition, we monitored the hydrodynamic diameter of Fe/SAE@A when incubated in both blood and FBS over a 96-h period. The results demonstrate that the particle size remained remarkably stable and consistent (approximately between 140 nm and 160 nm) throughout the entire incubation. This indicates excellent stability of our Fe/SAE@A in complex physiological environments, preventing aggregation or significant degradation (Fig. S3). The drug loading efficiency and release kinetics were measured. As shown in Fig. S4, the hydrophobic ADT can be effectively encapsulated into Fe/SAE. The release curves confirmed that ADT and H_2_S can efficiently release from in Fe/SAE under different pH (Fig. S5 and S6). These findings confirmed the successful synthesis of Fe/SAE@A.

**Fig. 2:**
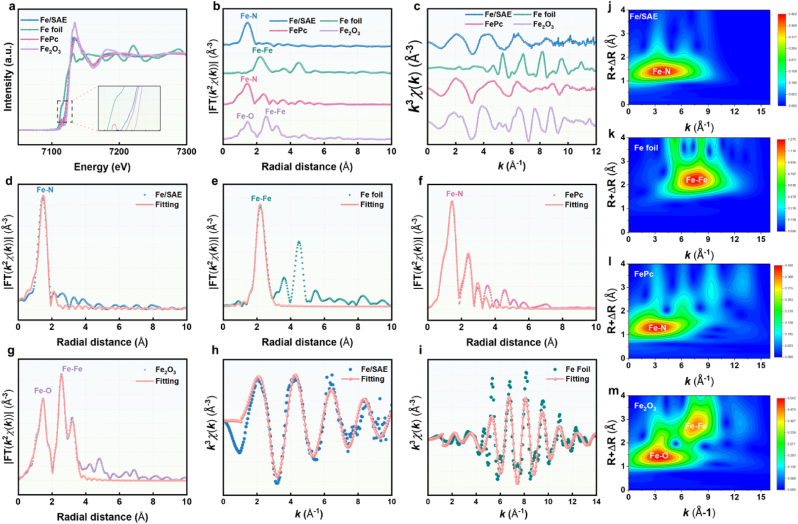
**Atomic structural analysis of Fe/SAE.** (a) XANES spectra of Fe/SAE. (b) EXAFS of the Fe/SAE. (c) EXAFS curves of Fe/SAE at the k space. (d) EXAFS fitting curves of Fe/SAE, (e) Fe foil, (f) FePc, and (g) Fe_2_O_3_ at the R space. (h) EXAFS fitting curve of Fe/SAE and (i) Fe foil at the k space. (j) Wavelet transformation of Fe/SAE, (k) Fe foil, (l) FePc, and (m) Fe_2_O_3_.

X-ray photoelectron spectroscopy (XPS) measurements were employed to evaluate the valence of C, Fe, and N atoms in Fe/SAE (Fig. S7). As illustrated in Fig. 1k, the C 1s spectrum of Fe/SAE mainly displayed characteristic corresponding to sp^2^-hybridized graphitic carbon (Fig. 1k). The N 1s spectrum unveiled three distinct nitrogen species: graphitic N, pyridinic N, and pyrrolic N. Notably, a substantial content of pyridinic nitrogen species was observed. This high content of pyridinic nitrogen is significant because it provides coordination sites for anchoring single-atomic Fe. This feature is crucial as it can enhance the catalytic activity of the Fe/SAE. (Fig. 1l). To confirm the coordination numbers of Fe, synchrotron radiation-based X-ray absorption near-edge structure (XANES) and extended X-ray absorption fine structure (EXAFS) analyses were conducted. The Fe K-edge XANES spectra exhibited an energy absorption threshold (Fig. 2a), confirming the Fe^δ+^ oxidation state, which was in agreement with the XPS results. Moreover, the Fourier transform of the EXAFS data in R-space revealed a prominent peak at 1.56 Å, corresponding to the Fe-N bond. Notably, no peaks were detected at 2.24 Å, which would have indicated Fe-Fe bonding. These results firmly established the presence of atomically dispersed Fe active sites within Fe/SAE (Fig. 2b–g). EXAFS fitting spectra at the Fe K-edge showed well-defined structures, demonstrating that the Fe atoms in Fe/SAE were coordinated with four N atoms (Fig. 2h–i, S8-S9, and Table S1). In addition, Fe/SAE exhibited a wavelet transform (WT) signal at 3.5 Å^−1^, corresponding to the Fe-N bond, with no WT intensity associated with Fe-Fe bonds (Fig. 2j–m). Collectively, these results demonstrated the successful construction of Fe/SAE.

**Fig. 3:**
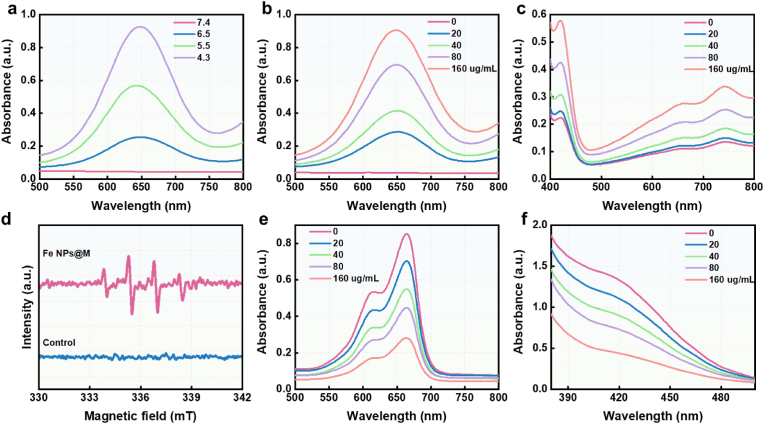
**Enzymatic performance of Fe/SAE.** (a) UV–vis spectra of TMB incubated with Fe/SAE plus H_2_O_2_ at various pH. (b) UV–vis spectra of TMB incubated with different concentrations of Fe/SAE plus H_2_O_2_. (c) The UV–vis spectra of ABTS treated with different concentrations of Fe/SAE in the presence of H_2_O_2_. (d) The ESR curves of •OH. (e) The UV–vis spectra of MB incubated with different concentrations of Fe/SAE plus H_2_O_2_. (f) UV–vis spectra of DTNB incubated with different concentrations of Fe/SAE in the presence of GSH.

We conducted a comprehensive evaluation of its enzymatic activities, focusing on its POD- and GSHOX-mimicking activities. To quantify the POD-mimicking activity of Fe/SAE, we employed the 3,3′,5,5′-tetramethylbenzidine (TMB) assay. When Fe/SAE was treated with H_2_O_2_, a rapid color change in TMB was observed at acidic pH levels (ranging from 4.3 to 6.5), but not at neutral pH (7.4). This observation indicates the pH-dependent catalytic behavior of Fe/SAE and its effective conversion of H_2_O_2_ into •OH (Fig. 3a and S10). Additionally, the enzymatic activity of Fe/SAE exhibited concentration-dependent manner (Fig. 3b and S11). To further confirm the generation of •OH, we used 2,2′-azino-bis(3-ethylbenzothiazoline-6-sulfonic acid) diammonium salt (ABTS). As shown in Fig. 3c and S12, Fe/SAE induced a notable increase in absorbance when ABTS was treated with H_2_O_2_, further demonstrating its excellent POD-like activity. To explore the product of •OH, we utilized dimethyl-1-pyrroline N-oxide (DMPO) and performed electron spin resonance (ESR) spectroscopy. ESR spectrum presented a quartet signal (1:2:2:1) of the DMPO/•OH (Fig. 3d). Moreover, methylene blue (MB) assays were conducted to assess •OH generation. The absorbance significantly decreased when MB was incubated with Fe/SAE and H_2_O_2_ (Fig. 3e and S13), indicating the exceptional ability of Fe/SAE to produce plentiful •OH. Furthermore, we conducted a TMB assay comparing Fe/SAE with other reported nanozymes (MnO_2_, Fe_2_O_3_, Co_2_O_4_). As shown in Fig. S14, Fe/SAE exhibited significantly higher POD-like activity, indicating its superior catalytic efficiency. We believe this comparison effectively addresses the reviewer's point. Fe/SAE exhibited superior POD-like activity compared to other single-atom nanozymes, including V/SAE, Co/SAE, and Ru/SAE (Fig. S15 and S6). Additionally, the GSHOX-like activity of Fe/SAE was evaluated using the 5,5′-dithiobis-(2-nitrobenzoic acid) (DTNB) probe. The absorbance at 412 nm significantly decreased in a concentration-dependent manner, confirming the remarkable GSH depletion capacity of Fe/SAE (Fig. 3f and S17). Collectively, these results suggest that Fe/SAE exhibits excellent catalytic performances and holds great promise for boosting ROS cascades.

### 2.2 Therapeutic efficacy of Fe/SAE@A in cancer cell

**Fig. 4:**
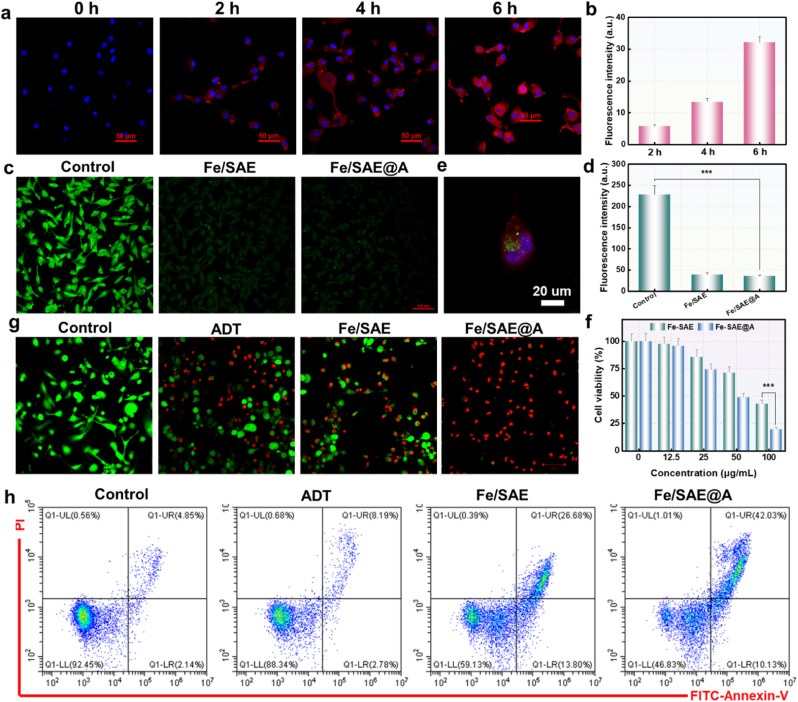
**The therapeutic efficacy on tumor cells.** (a) Fluorescence images and (b) corresponding quantification. (c) Fluorescence images and (d) corresponding quantification of calcein-AM-stained. (e) CLSM images of GL261 cells colocalization. (f) CCK-8 assay. (g) CLSM images of live/dead staining. (h) Flow cytometry measurements. Data are presented as mean ± standard deviation (n = 3).

Building upon its exceptional catalytic activity, the antitumor efficacy of Fe/SAE@A was assessed. To evaluate the uptake behavior, we utilized a cyanine 5.5 (Cy5.5)-labeled Fe/SAE@A. Confocal laser scanning microscopy (CLSM) imaging revealed a time-dependent enhancement, indicating progressive internalization of Fe/SAE@A over time (Fig. 4a and b and S18). Additionally, intracellular Fe^2+^ levels were quantified using calcein-AM. Upon entering the cells, calcein-AM is metabolized by intracellular esterases into the fluorescent calcein. The fluorescence of calcein is subsequently quenched upon interaction with Fe/SAE@A (Fig. 4c and d). The results from a colocalization experiment further demonstrated that Fe/SAE@A predominantly localized in lysosomes and the cytoplasm (Fig. 4e and S19), consistent with the acidic conditions required to facilitate the Fe/SAE@A-mediated Fenton reaction. To assess the antitumor efficacy, a Cell Counting Kit-8 (CCK-8) assay was performed, showing a significant reduction in GL261 cell survival with increasing concentrations of Fe/SAE (Fig. 4f). Importantly, the survival rate of tumor cells treated with Fe/SAE@A was lower than that of the Fe/SAE group, likely due to H_2_S inhibition of ROS-scavenging enzymes, which substantially enhanced the ROS cascade. Furthermore, propidium iodide (PI)/calcein-AM co-staining was conducted to measure the cell killing effect. Tumor cells incubated with Fe/SAE revealed a markedly higher rate of apoptosis. Moreover, the cytotoxicity of Fe/SAE@A on tumor cells was further amplified by the addition of H_2_S (Fig. 4g and S20). Additionally, flow cytometry results revealed a remarkably higher percentage of cell death in Fe/SAE-treated cells in comparison to PBS. Notably, the cancer cell death rate was further enhanced by the cooperation of Fe/SAE plus H_2_S therapy (Fig. 4h and S21).These findings provided strong evidences that Fe/SAE@A effectively suppresses tumor proliferation by exploiting its H_2_S-boosted catalytic performance.

**Fig. 5:**
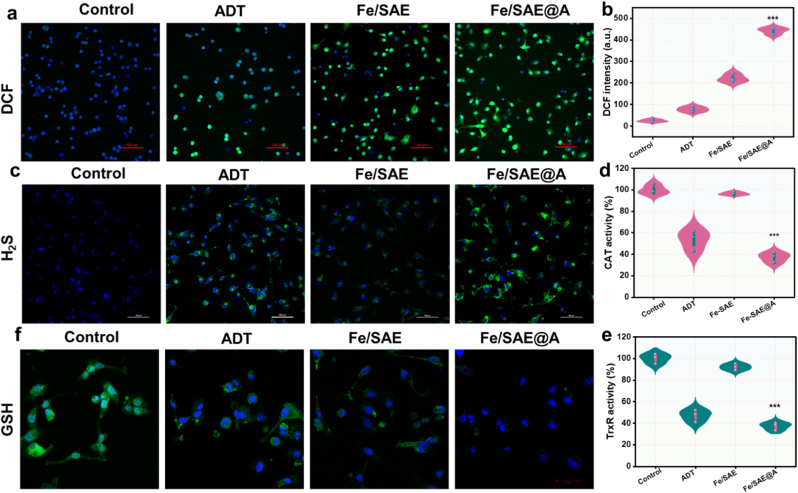
**The ROS generation and H_2_S release in tumor cells.** (a) DCF fluorescence images and (b) corresponding quantification. (c) H_2_S fluorescence images and (b) corresponding quantification. (d) The CAT and (e) TrxR activity in tumor cells. (f) GSH fluorescence images of cancer cells. Data are presented as mean ± standard deviation (n = 3).

The anti-tumor mechanism of Fe/SAE@A was subsequently investigated to gain a comprehensive understanding of its therapeutic effects. Given the POD-like activity of Fe/SAE@A and its H_2_S-amplified ROS cascade, 2′,7′-dichlorofluorescein diacetate (DCFH-DA) was utilized to evaluate ROS level. CLSM images demonstrated that Fe/SAE@A significantly elevated ROS levels, with H_2_S further enhancing ROS accumulation in GL261 cells (Fig. 5a and b and S22). To evaluate intracellular H_2_S release, the H_2_S-specific fluorescent probe, Washington State Probe-5 (WSP-5), was employed. Confocal laser scanning microscopy (CLSM) images confirmed that both ADT and Fe/SAE@A were highly effective in promoting H_2_S production (Fig. 5c and S23), which in turn significantly inhibited the activity of TrxR and CAT (Fig. 5d and e). In addition, we studied the CAT and TrxR mRNA levels using a qRT-PCR assay. As shown in Fig. S24 and S25, the CAT and TrxR mRNA levels showed no remarkable change. In addition, bismuth subsalicylate treatment can effectively enhance the CAT and TrxR activity, confirming the H2S scavenging can rescue the enzymatic activity. Moreover, intracellular GSH plays a critical role in buffering ROS levels. Notably, Fe/SAE@A exhibited a remarkable capacity to deplete GSH, inducing oxidative stress and triggering a ROS storm within the cells (Fig. 5f and S26-27). Collectively, these findings confirm that the ROS generation, H_2_S delivery, and GSH consumption ability of Fe/SAE@A substantially contribute to tumor cell death.

**Fig. 6:**
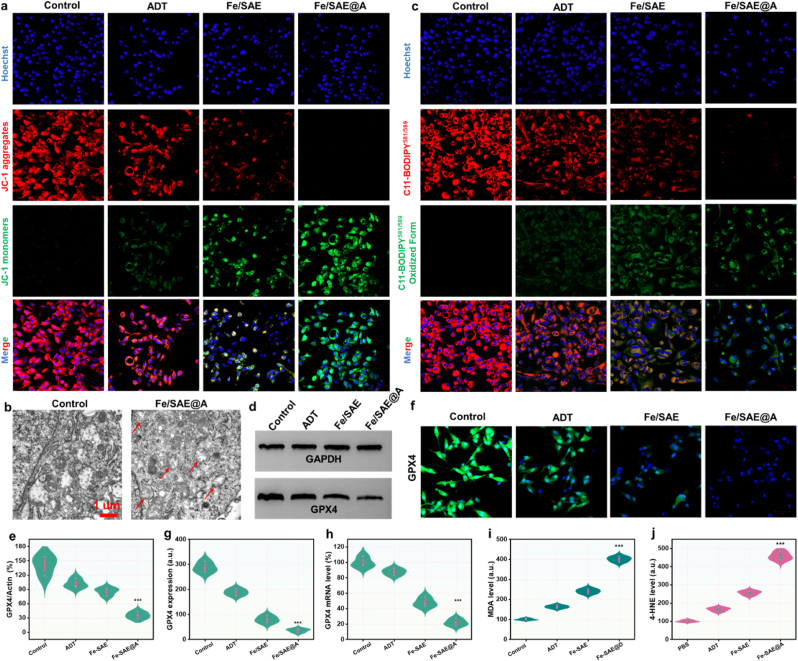
**Mechanism of Fe/SAE induced tumor cells ferroptosis.** (a) CLSM images of JC-1 fluorescence. (b) Bio-TEM of GL261 cells. (c) Fluorescence images of fluorescent probe C11-BODIPY^581/589^. (d) GPX4 expression and (e) corresponding quantification by WB. (f) GPX4 expressions and (g) corresponding quantification by immunofluorescence assay. (h) The GPX4 mRNA level in GL261 cells. (i) MDA and (j) 4-HNE level in GL261. Data are presented as mean ± standard deviation (n = 3).

To assess mitochondrial membrane potential polarization, we utilized the mitochondria-specific probe JC-1, which monitors the shift in fluorescence from red to green. As demonstrated in the CLSM images (Fig. 6a), the green fluorescence intensity in the Fe/SAE was markedly brighter compared to the PBS group. The release of H_2_S further intensified the green fluorescence in the Fe/SAE@A group, while the red fluorescence displayed an inverse trend. Additionally, biological transmission electron microscopy (Bio-TEM) analysis of GL261 cells treated with Fe/SAE@A revealed mitochondrial damage, a key characteristic of ferroptosis (Fig. 6b). These observations indicated mitochondrial membrane potential polarization in cancer cells, implying that Fe/SAE@A may trigger ferroptosis. To further validate this, we examined key indicators of ferroptosis. To monitor LPO accumulation in the cell membranes, we utilized the fluorescent indicator C11-BODIPY^581/589^. Fe/SAE treatment significantly enhanced green fluorescence while progressively reducing red fluorescence, a clear sign of LPO accumulation. This effect was further intensified in the presence of ADT-released H_2_S (Fig. 6c). In parallel, western blot (WB) analysis revealed that Fe/SAE@A treatment led to a downregulation of GPX4 expression (Fig. 6d–e), a finding supported by both immunofluorescence and qRT-PCR data (Fig. 6f–h and S28). Furthermore, we measured ferroptosis markers, including malondialdehyde (MDA) and 4-hydroxynonenal (4-HNE). The combination of Fe/SAE and ADT caused a marked increase in MDA and HNE levels (Fig. 6i and j). Furthermore, we detect the key ferroptosis markers such as ACSL4, SLC7A11 using an immunofluorescence assay. As shown in Fig. S29 and S30, experimental results demonstrated that Fe/SAE@A can effectively upregulate the ACSL4 expression and downregulate the SLC7A11 expression. Furthermore, the ferroptosis inhibitor Ferrostatin-1 efficiently rescue the Fe/SAE@A-induced cell death, as convinced in CCK-8 results (Fig. S31). Collectively, these results demonstrate that Fe/SAE@A induces ferroptosis through LPO accumulation and the inactivation of GPX4.

### 2.3 In vivo antitumor efficacy of Fe/SAE@A

**Fig. 7:**
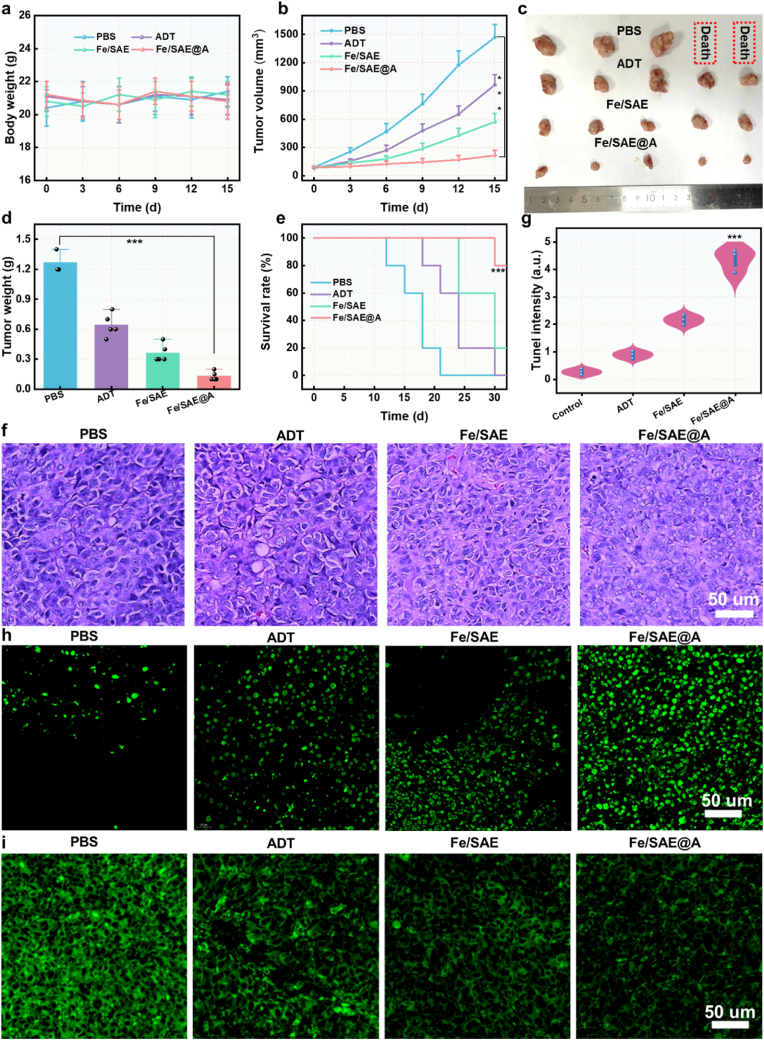
**Antitumor efficacy of Fe/SAE@A.** (a) The body weight curves of mice. (b) The tumor volume curves of mice. (c) The photographs and (d) tumor weights. (e) The Kaplan-Meier survival curves of mice. (f) H&E staining, (h) GPX4 immunofluorescence, and (i) ROS immunofluorescence of tumors following various formulations. Data are presented as mean ± standard deviation (n = 5). Statistical analyses were performed using the Student's two-tailed *t*-test (∗P < 0.05, ∗∗P < 0.01, ∗∗∗P < 0.001).

The biocompatibility of Fe/SAE@A was evaluated before assessing its therapeutic efficacy in mice. Hematoxylin and eosin (H&E) staining was conducted to study the morphology of various tissues, showing no significant histological damage (Fig. S32). In addition, the biochemical factors were measured. As show in Fig. S33, the biochemical factors showed no significant change following Fe/SAE@A injection. We investigate biodistribution using an ICP-MS. Results showed the Fe/SAE@A can effectively accumulate at the tumor site and be metabolized out by the liver (Fig. S34). Given its H_2_S delivery capabilities and impressive catalytic performances, antitumor efficacy of Fe/SAE@A was further investigated. GL261-bearing nude mice were assigned to four treatment groups: Control, ADT, Fe/SAE, and Fe/SAE@A. Body weight measurements indicated no significant weight changes in either the Fe/SAE or Fe/SAE@A groups (Fig. 7a), further supporting the excellent biocompatibility of Fe/SAE@A. Tumor growth progression was closely measured, with the results depicted in Fig. 7b. Treatment with Fe/SAE, which induces ferroptosis via the inactivation of GPX4 and the accumulation of LPO, partially inhibited tumor growth. Notably, the Fe/SAE@A group exhibited a higher antitumor effect, attributed to its unique H_2_S-enhanced ROS cascade (Fig. 7c and d). Kaplan-Meier survival analysis revealed that Fe/SAE@A significantly improved survival rates, demonstrating substantial therapeutic efficacy against tumors (Fig. 7e). The antitumor effect of Fe/SAE@A was confirmed via H&E and Terminal deoxynucleotidyl transferase dUTP nick end labeling (TUNEL) staining. H&E staining demonstrated extensive tumor tissue damage in mice treated with Fe/SAE@A, highlighting the potent therapeutic effect of Fe/SAE@A (Fig. 7f). Additionally, TUNEL staining indicated the highest levels of cell death in the Fe/SAE@A treatment group (Fig. 7f–g and S35). Immunofluorescence staining further demonstrated an obvious downregulation of GPX4 following Fe/SAE@A treatment (Fig. 7h and S36). ROS levels were markedly elevated in the tumor cells of the Fe/SAE@A-treated group, attributed to the H_2_S-mediated inhibition of ROS-scavenging enzymes and the POD- and GSHOX-like catalytic activities of Fe/SAE (Fig. 7i and S37). Collectively, these findings underscore the role of Fe/SAE@A in releasing H_2_S, generating •OH, consuming GSH, and accelerating LPO accumulation, ultimately leading to tumor ferroptosis.

## 3 Conclusion

In summary, we have developed the Fe/SAE system as a H_2_S delivery platform to potentiate ROS-driven ferroptosis in cancer cells. Fe/SAE, featuring atomically dispersed active metal sites, demonstrated robust catalysis resembling POD- and GSHOX-activities, promoting •OH generation from H_2_O_2_ and depletion of GSH. Furthermore, the system facilitated H_2_S-induced inhibition of ROS-scavenging enzymes, within the TME. Upon internalization by cancer cells, Fe/SAE@A triggered H_2_S-mediated inactivation of TrxR and CAT, thereby elevating intracellular H_2_O_2_ levels and initiating a potent ROS storm in GL261 cells, ultimately leading to irreversible LPO. Additionally, Fe/SAE@A effectively depleted endogenous reductive GSH, further activating GPX4 inactivation. Comprehensive in vitro and in vivo investigations revealed the underlying mechanism of tumor cell death mediated by Fe/SAE@A, highlighting its dual role in H_2_S delivery, ROS cascade initiation, and reductive GSH depletion, all of which induce LPO-driven ferroptosis. This study presents a promising antitumor strategy by exploiting the ferroptosis pathway to deliver H_2_S, inactivate GPX4, and enhance LPO accumulation.

## CRediT authorship contribution statement

**Xuegang Niu:** Writing – review & editing, Writing – original draft, Conceptualization. **Penghui Wei:** Data curation. **Mingtao Zhu:** Formal analysis. **Hongjia Zheng:** Formal analysis. **Dezhi Kang:** Investigation. **Qianxi Chen:** Data curation. **Fuxiang Chen:** Methodology. **Yiping Li:** Data curation. **Rong Xie:** Methodology. **Yang Zhu:** Writing – review & editing, Writing – original draft, Supervision. **Dengliang Wang:** Resources.

## Ethics approval and consent to participate

Animal experiments were performed according to the protocol approved by The Ethical Committee of Fujian Medical University (IACUC FJMU2022-0608).

## Consent for publication

All authors agreed to submit this manuscript.

## Funding declaration

This work was supported by the Joint Funds for the innovation of science and Technology, Fujian province (NO.2024Y9163, NO.2024Y9137, NO.2023Y9090), Beijing Postdoctoral Research Funding, 10.13039/501100017686Fujian Provincial Health Technology Project (NO.2024CXA017, No.2024QNA030), the Natural Science Foundation of Nanping City of China (N2021J050).

## Declaration of competing interest

The authors declared that they have no known competing financial interests or personal relationships that could have appeared to influence the work reported in this paper.

## Data Availability

No data were used for the research described in the article.
