# Supplementary material for: Iron single atom enzyme-mediated hydrogen sulfide delivery amplifies reactive oxygen species cascade to induce ferroptosis susceptibility
Source: Mater Today Bio. 2025 Aug 9;34:102184. doi: 10.1016/j.mtbio.2025.102184 (PMC12363588; doi:10.1016/j.mtbio.2025.102184)
Supplement: Multimedia component 1 [file mmc1.docx]

**Supporting Information**

**Chemicals**

2,2’-azino-bis(3-ethylbenzothiazoline-6-sulfonic acid) diammonium salt (ABTS), ironylacetonate (Fecac)_3_), hydrogen peroxide (H_2_O_2_), acetic acid (HAc), sodium acetate (NaAc), and ethanol were purchased from Sinopharm Chemical Reagents (Shanghai, China). 3,3',5,5'-tetramethylbenzidine (TMB), anethole trithione (ADT) and C11-BODIPY^581/591^ were provided by Sigma-Aldrich (St. Louis, USA). Hoechst 33342, 2′,7′-dichlorofluorescin diacetate (DCFH-DA), cell count kit-8 (CCK-8), annexin V-FITC/PI apoptosis detection kit, thiobarbituric acid (TBA), and 1,1',3,3'-tetraethyl-5,5',6,6'-tetrachloroimidacarbocyanine iodide (JC-1) were bought from Beyotime (Shanghai, China). Dulbecco's modified eagle medium (DMEM) was purchased from Hyclone (Logan, USA). 5,5-dimethyl-1-pyrroline N-oxide (DMPO) was bought from Dojindo (Dojindo). Cyanine 5.5 monosuccinimidyl ester (Cy5.5-NHS), ELISA, and annexin V-FITC/PI apoptosis detection kit were purchased from Beijing Solarbio Science & Technology Co., Ltd. (Beijing, China). Live & Dead Staining Kit (Cat#40274ES60) and GMyc-PCR Mycoplasma Test Kit (Cat#40601) was purchased from Yeasen Biotechnology (Shanghai) Co., Ltd.. 20 mm glass-bottom dishes, and centrifuge tubes were obtained from NEST Biotechnology Co. Ltd. (Wuxi, China). Deionized (DI) water was obtained from a Milli-Q water purification system.

**Instruments**

Powder X-ray diffraction (XRD) patterns were recorded on a Rigaku Miniflex-600 diffractometer. Transmission electron microscope (TEM) images were taken by Hitachi-7700. High-angle annular dark field scanning transmission electron microscopy (HAADF-STEM) images were recorded by JEM-ARM200F (JEOL) TEM/STEM with a spherical aberration corrector. The energy-dispersive X-ray spectroscopy (EDS) mapping was performed by JEM-2100F. X-ray photoelectron spectroscopy (XPS) spectra were collected on scanning X-ray microprobe (PHI 5000 Verasa, ULAC-PHI). Scanning electron microscopy (SEM) images were taken by Nova NanoSEM 230. Fluorescence imaging was performed by confocal microscopy (Nikon C2). The absorption spectra were measured by a ultraviolet-visible (UV-vis) UH4150 spectrophotometer (Hitachi). Metal content was measured by using inductively coupled plasma mass spectrometer (ICP-MS, PlasmaQuad 3, Thermo Elemental). Hydrodynamic diameters and zeta potentials were determined by a Zetasizer nano ZS instrument (Malvern). Cancer cell apoptosis was monitored by using a flow cytometer (CytoFLEX, Beckman).

**Experimental section**

**Synthesis of Fe-N_4_/SAE and Fe-S_1_N_3_/SAE**

Fe@ZIF-8 precursors were synthesized using a host-guest strategy. 3.54 g 2-methylimidazole and 100 mg Fe(acac)_3_ were added into a 150 mL flask containing 60 mL methanol as solution A. 1.6 g Zn(NO_3_)_2_•6H_2_O was dissolved in 60 mL methanol as solution B. Then solution A was mixed with solution B, and the mixture was further stirred for 12 h. Then the Fe@ZIF-8 powders were collected by centrifugation, washed with methanol several times, and dried at 65 °C in a vacuum oven overnight. And the Fe-N_4_/SAE was obtained by the one-step pyrolysis strategy. Firstly, the Fe@ZIF-8 powder were placed successively in a tubular furnace, and then pyrolysis at 950 ºC for 3 h in an argon atmosphere. 10 mg Fe-N_4_/SAE and 10 mL 10 mg/mL DSPE-PEG were mixed and ultrasound for 20 min, then 1.5 mL 1 mg/ mL ADT (in DMSO) was added. the mixture was further stirred for 12 h and the product Fe/SAE@A were collected by centrifugation and washed three time with PBS.

**•OH generation by Fe-N_4_/SAE-mediated catalytic reaction**

The catalytic activity of Fe-N_4_/SAE were measured by using TMB as a probe which can be converted into oxidized TMB with blue color by •OH. Briefly, Different concentrations of Fe-N_4_/SAE and H_2_O_2_ (100 μM) were successively added into the PBS solutions with TMB (40 ug/mL), and the mixtures were shaken at 37 °C for 10 min. After centrifugation, the absorption spectra of supernatant were measured.

Fe-N_4_/SAE and H_2_O_2_ (10 mM) were mixed with MB (20 ug/mL) solutions at acidic pH, and the mixtures were shaken at 37 °C for 10 min. After ultrafiltration, the •OH-induced MB degradation was measured by the absorbance change at 652 nm.

Electron spin resonance (ESR) analysis was carried out using DMPO as the spin trapper. To confirm the Fe-N_4_/SAE-mediated •OH generation. 10 mM NaAc-HAc buffer solution (pH 4.3) containing 5 mM H_2_O_2_, Fe-N_4_/SAE (50 μg/mL), and 100 mM DMPO was ultrasonicated for 1 min. Then, the mixture was transferred to a quartz tube for ESR measurement.

**GSH consume by Fe-N_4_/SAE mediated catalytic reaction**

Fe-N_4_/SAE were mixed with GSH (10 mM) solutions at acidic pH, and the mixtures were shaken at 37 °C for 30 min. Finally, the DTNB (0.5 mg/ml) solution was added. After ultrafiltration, the GSH depletion was measured by the absorbance change at 415 nm.

**Cytotoxicity assessments**

Cell-viability was determined by the CCK-8 assay. For CCK-8 assay, GL261 cells were planted for 24 h. Then, the cells were incubated with various concentrations of Fe/SAE@A. After treatment for 24 h, the medium was replaced with fresh medium containing 10 μL CCK-8 and quantified by the absorbance at 450 nm using a microplate reader. For analysis of cell death, Annexin V-FITC and PI kit was conducted. GL261 cells were seeded and incubated 12 h. Subsequently, the cells were exposed to Fe/SAE@A. After co-staining with Annexin V-FITC and PI according to the manufacturer’s protocols. The quantitative cell death was analyzed by flow cytometry.

**In vitro ROS generation**

GL261 cells were seeded in confocal dish. After incubation for 12 h, the cells were treated with various formulation for 4 h. Then, the cells were co-stained with DCFH-DA (10 μM) and Hoechst (20 μM). After 20 minutes of incubation, the fluorescence imaging of cells was imaged by confocal microscopy.

**Analysis of the change of mitochondrial membrane potential (MMP)**

To investigate the MMP, GL261 cells were seeded and incubated for 24 h. Subsequently, the cells were exposed to Fe/SAE@A. Then the cells were treated according to the JC-1 kit. The fluorescence imaging of cells was analyzed by confocal microscopy.

**LPO initiated by Fe-N_4_/SAE@E**

The cellular LPO assay was carried out by using a BODIPY^581/591^-C11 probe. GL261 cells were seeded and incubated for 24 h. Subsequently, the cells were exposed to Fe/SAE@A. Then the cells were stained with BODIPY^581/591^-C11 probe and Hoechst for 20 min. The fluorescence imaging of cells was imaged by CLSM.

**Analysis of the change of MDA**

To investigate the MDA, GL261 cells were seeded and incubated for 24 h. Subsequently, the cells were exposed to Fe/SAE@A. Then the cells were treated according to the MDA kit. The assay was carried out according to the manufacturer’s instructions.

**In vivo antitumor efficacy**

Animal experiments were performed according to the protocol approved by The Ethical Committee of Fujian Medical University. The right hind legs of all mice were subcutaneously transplanted with GL261 cells (1 × 10^6^ cells suspended in 100 μL of PBS). The tumor-bearing mice were used for antitumor treatment until the tumor volume reached about 70 mm^3^. GL261 tumor-bearing nude mice were randomly divided into 4 groups (5 mice per group): Fe/SAE@A, Fe/SAE, ADT or PBS. The mice were administered 5 mg/kg intravenously every two days for a total of three doses. The tumor volumes and body weights were recorded every three days. After 15 days of treatment, one mice from each group were euthanatized for histological examination and immunofluorescence.

**Statistical analysis**

All quantitative data were expressed as the mean ± standard deviation (SD). Statistical analyses were performed using the Student’s two-tailed t-test (*P < 0.05, **P < 0.01, ***P < 0.001).

**
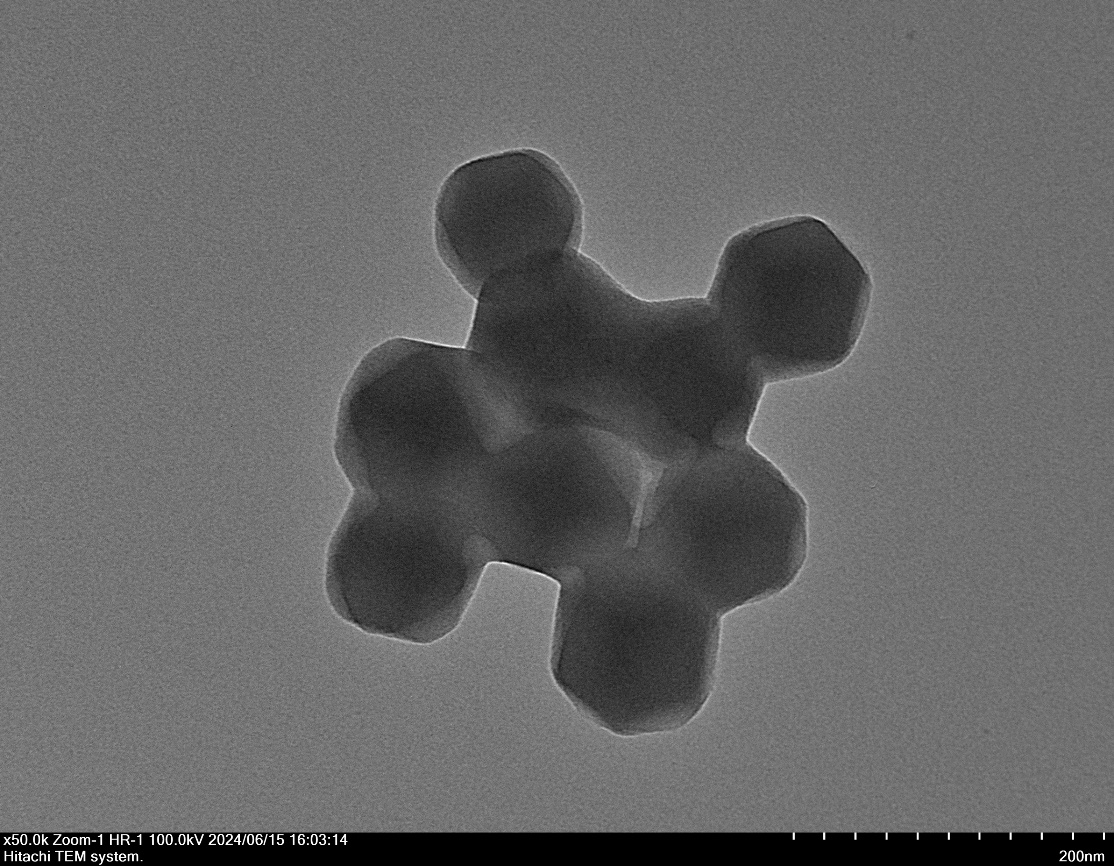
**

**Figure S1.** The TEM images of Mn@ZIF-8.


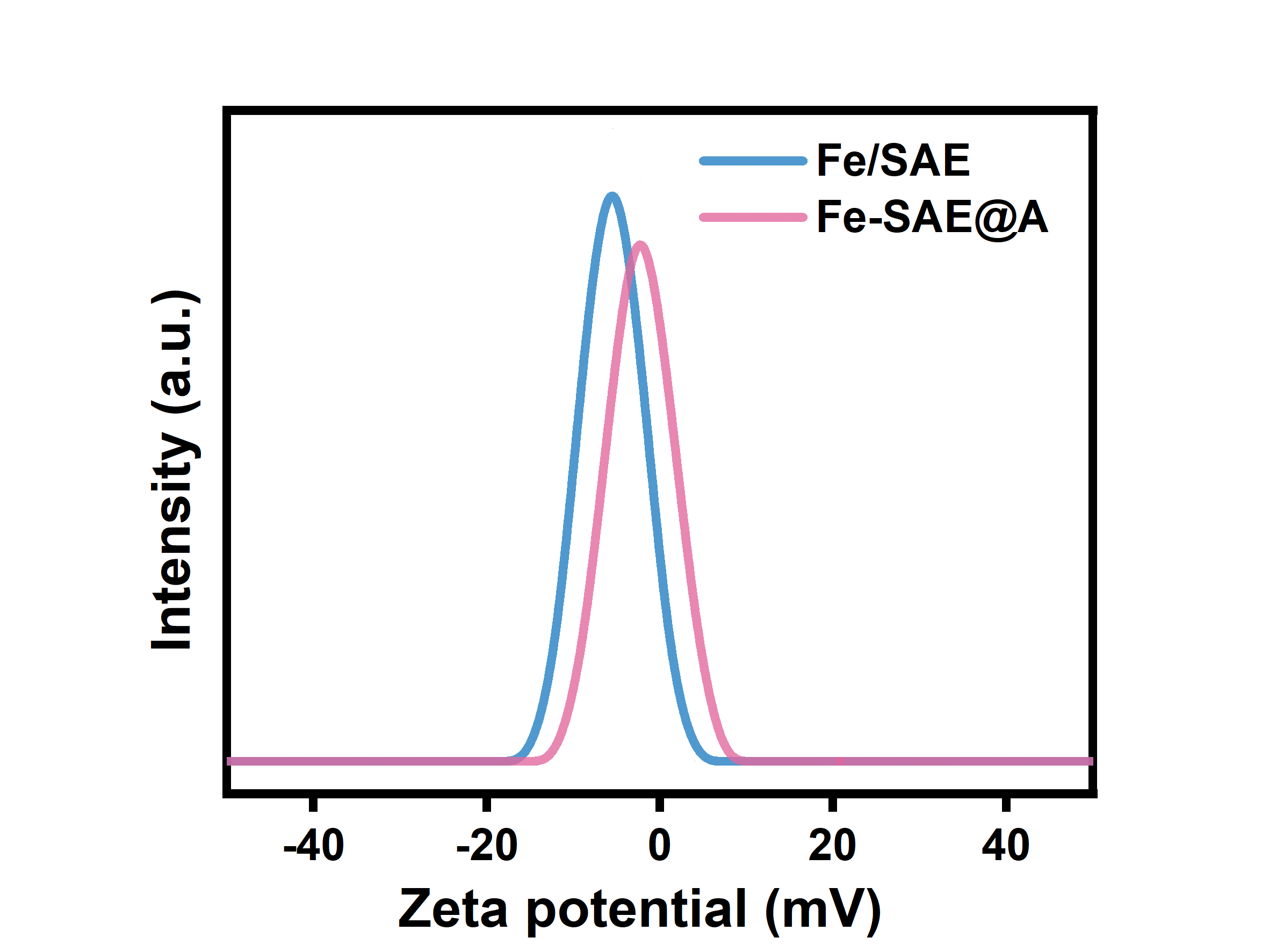


**Figure S2**. Zeta potential distribution of Fe/SAE and Fe/SAE@A.


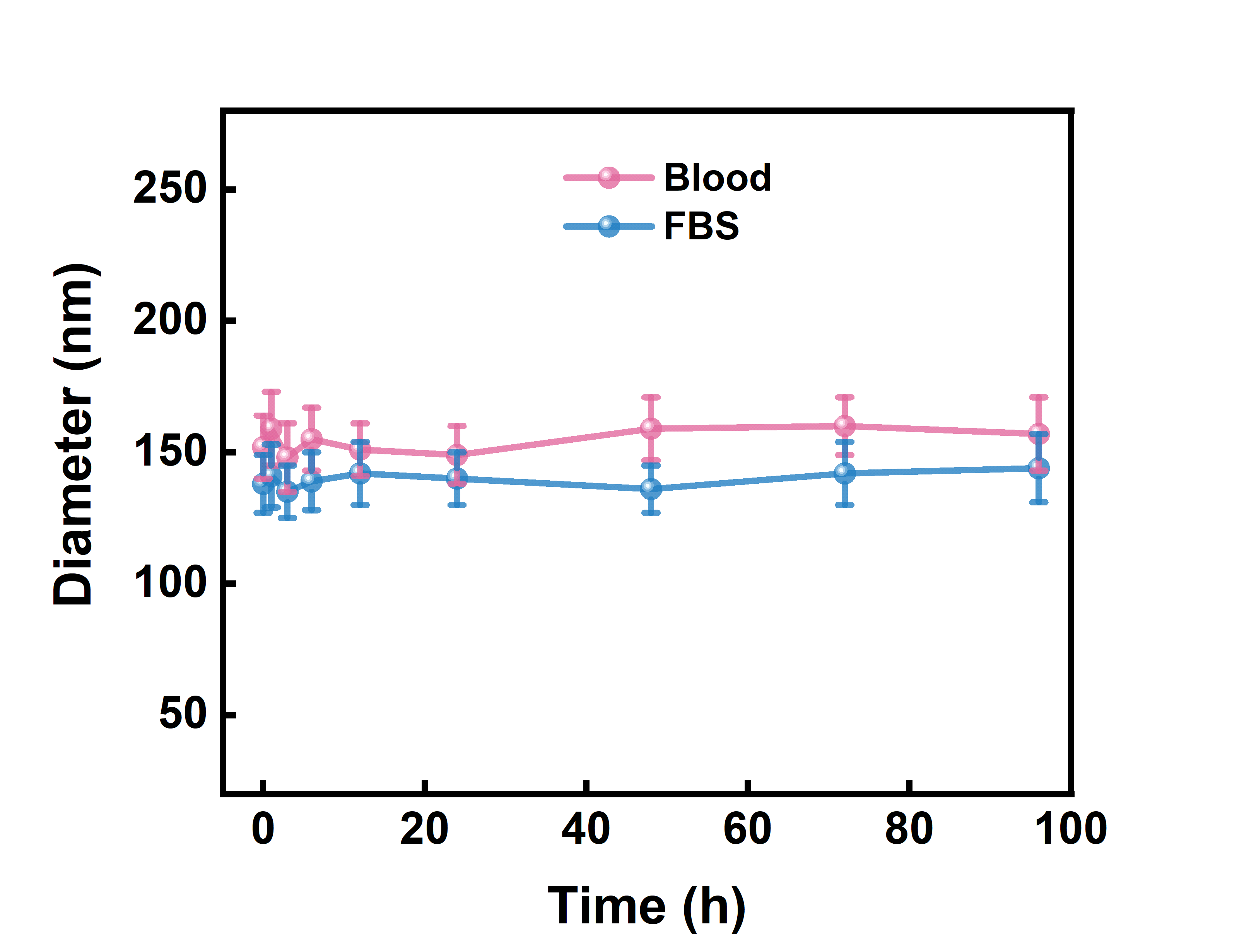


**Figure S3.** Hydrodynamic diameter of Fe-SAE@D NPs) incubated in blood and fetal bovine serum (FBS) over 96 hours. Data are presented as mean ± standard deviation (n=3).


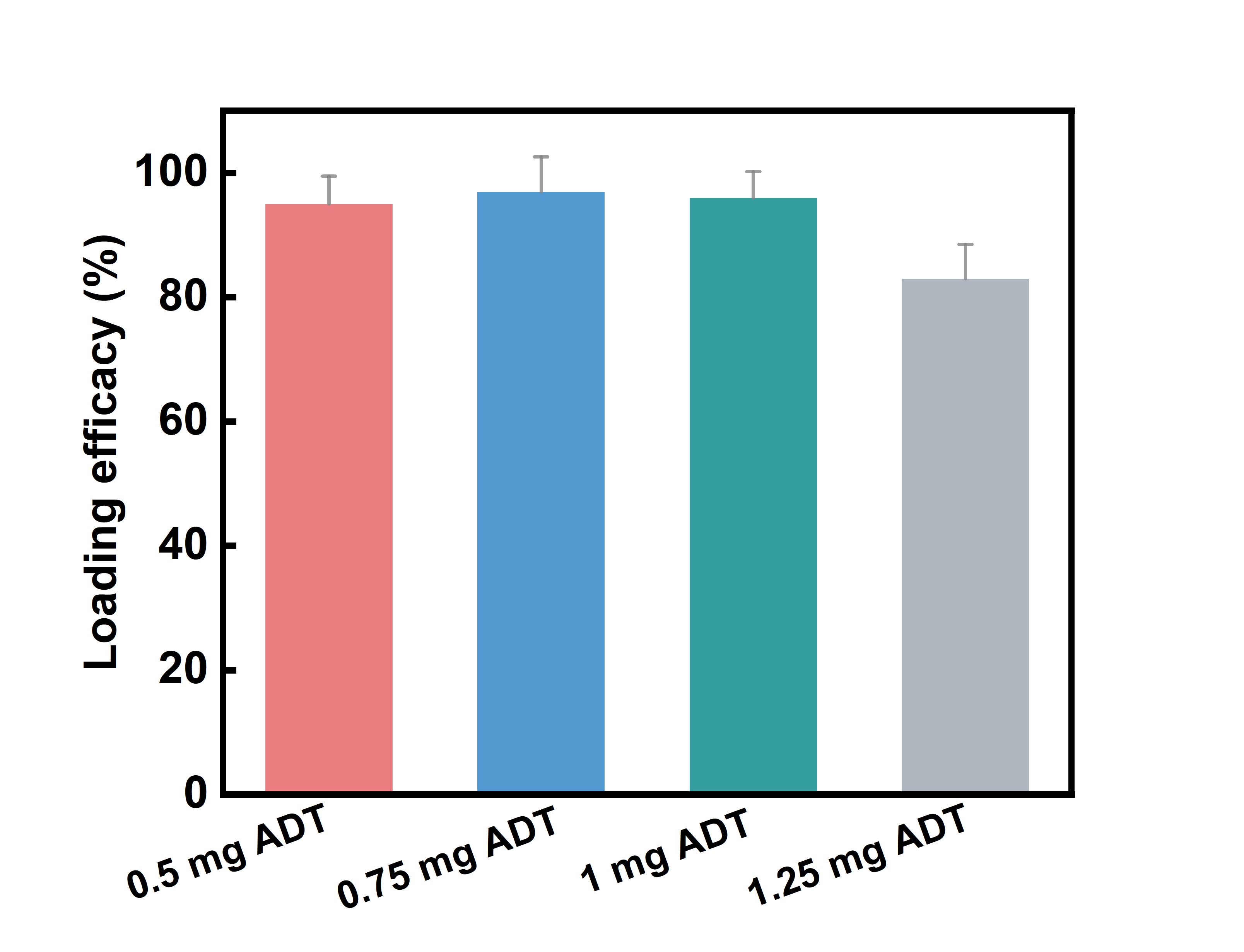


**Figure S4.** The load efficacy of Fe/SAE@A after adding different quality of ADTs.


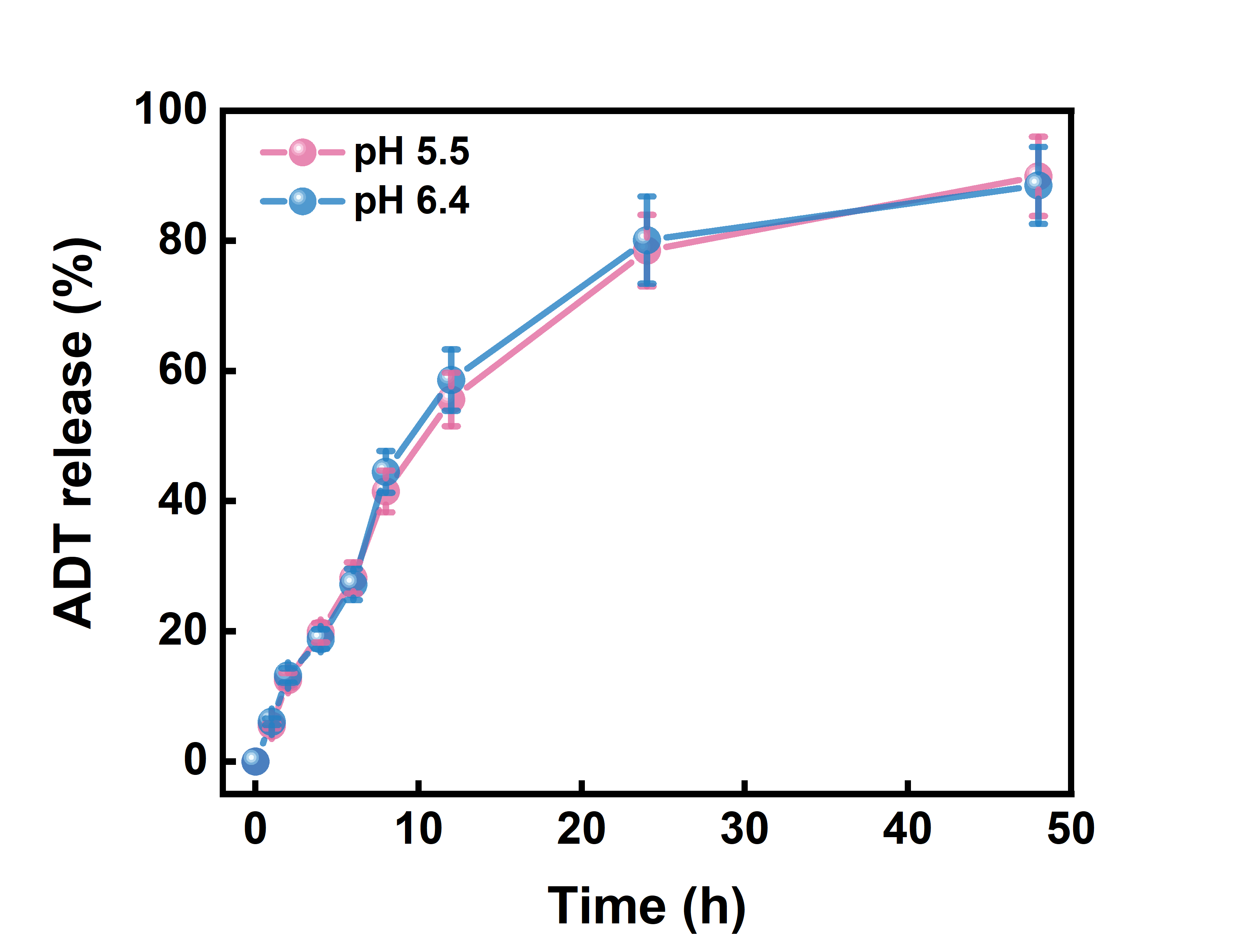


**Figure S5.** The release of ADT from Fe/SAE@A under different pH.


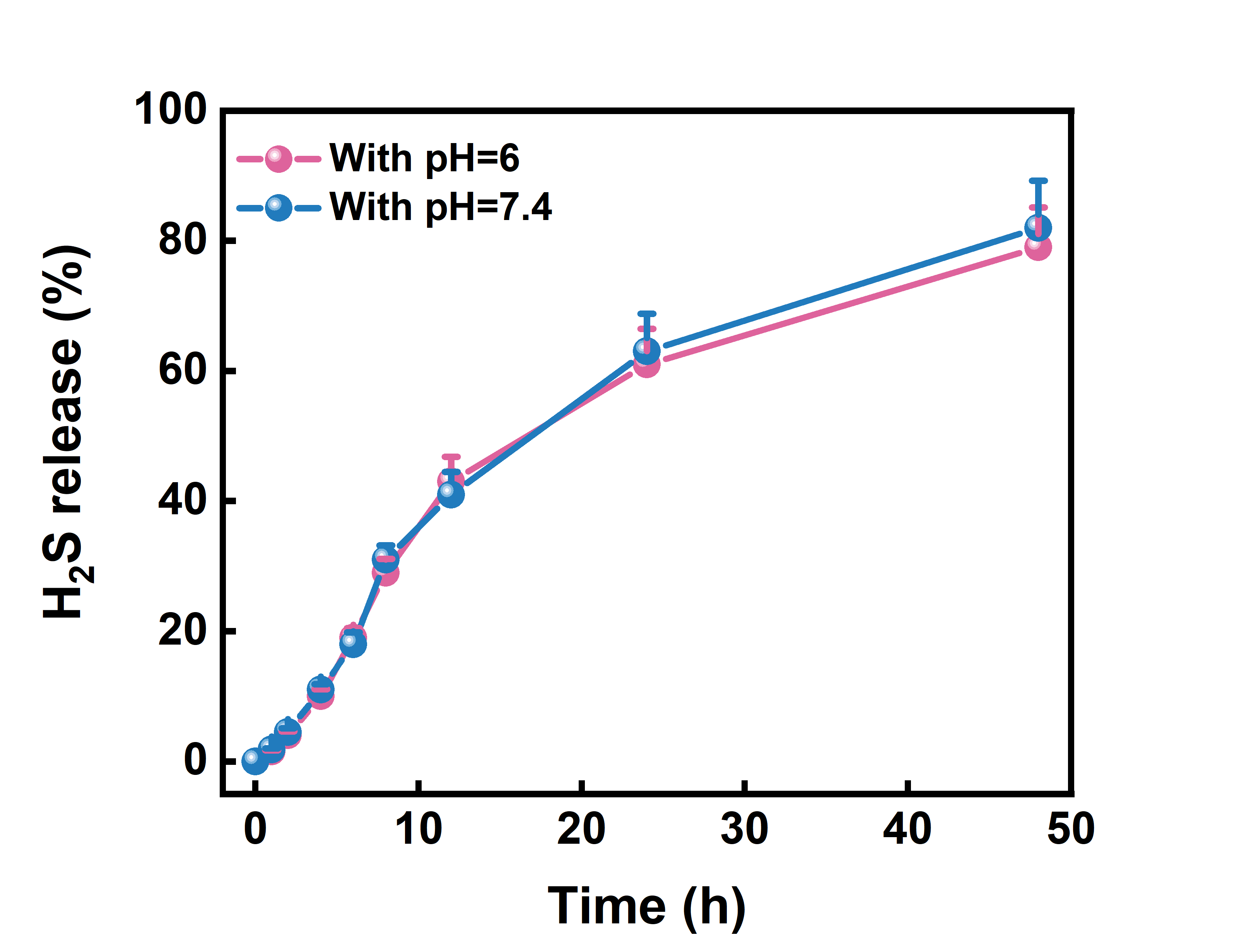


**Figure S6.** The H_2_S release curves in a time-dependent manner under different pH.


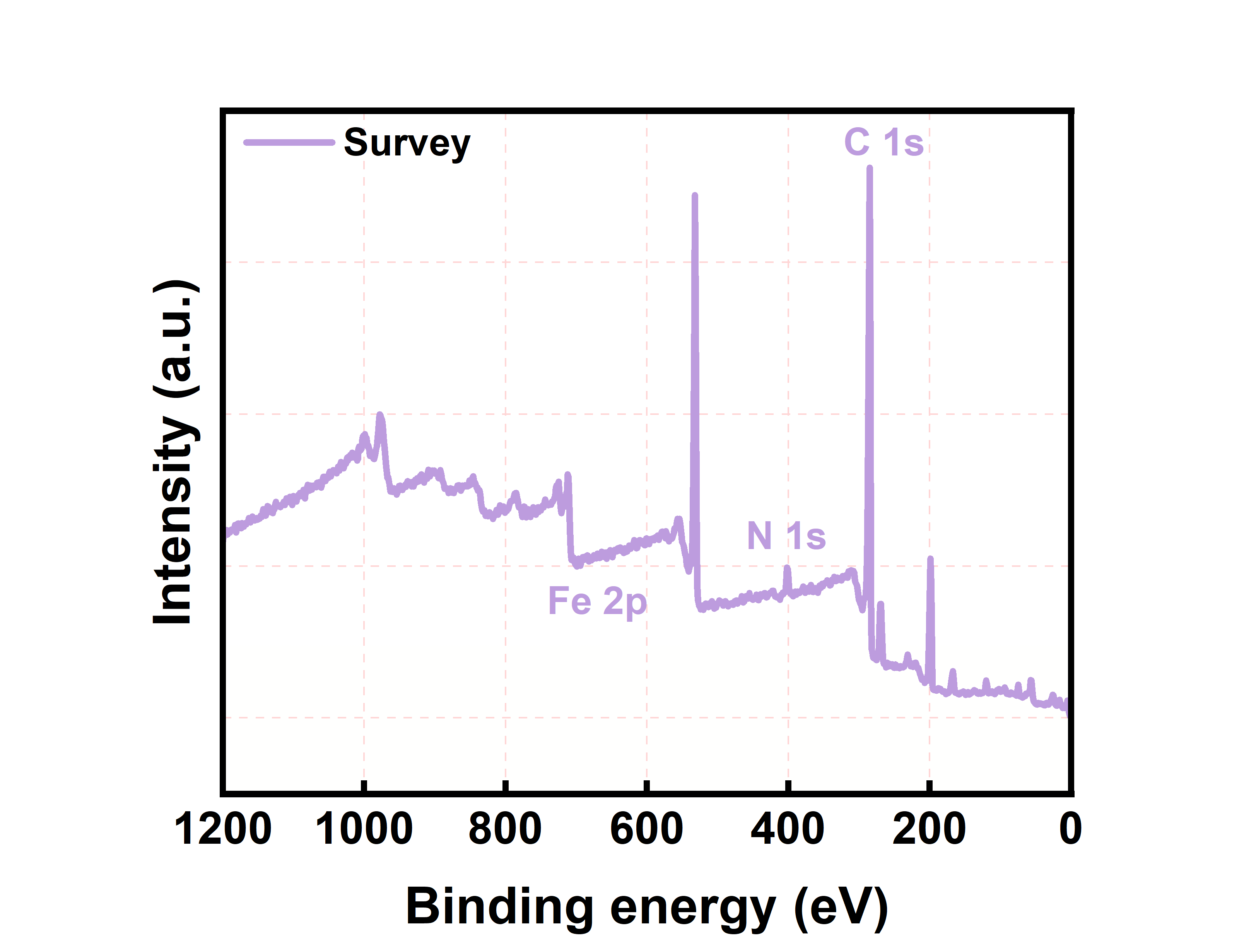


**Figure S7.** The survey XPS spectrum of Fe/SAE.


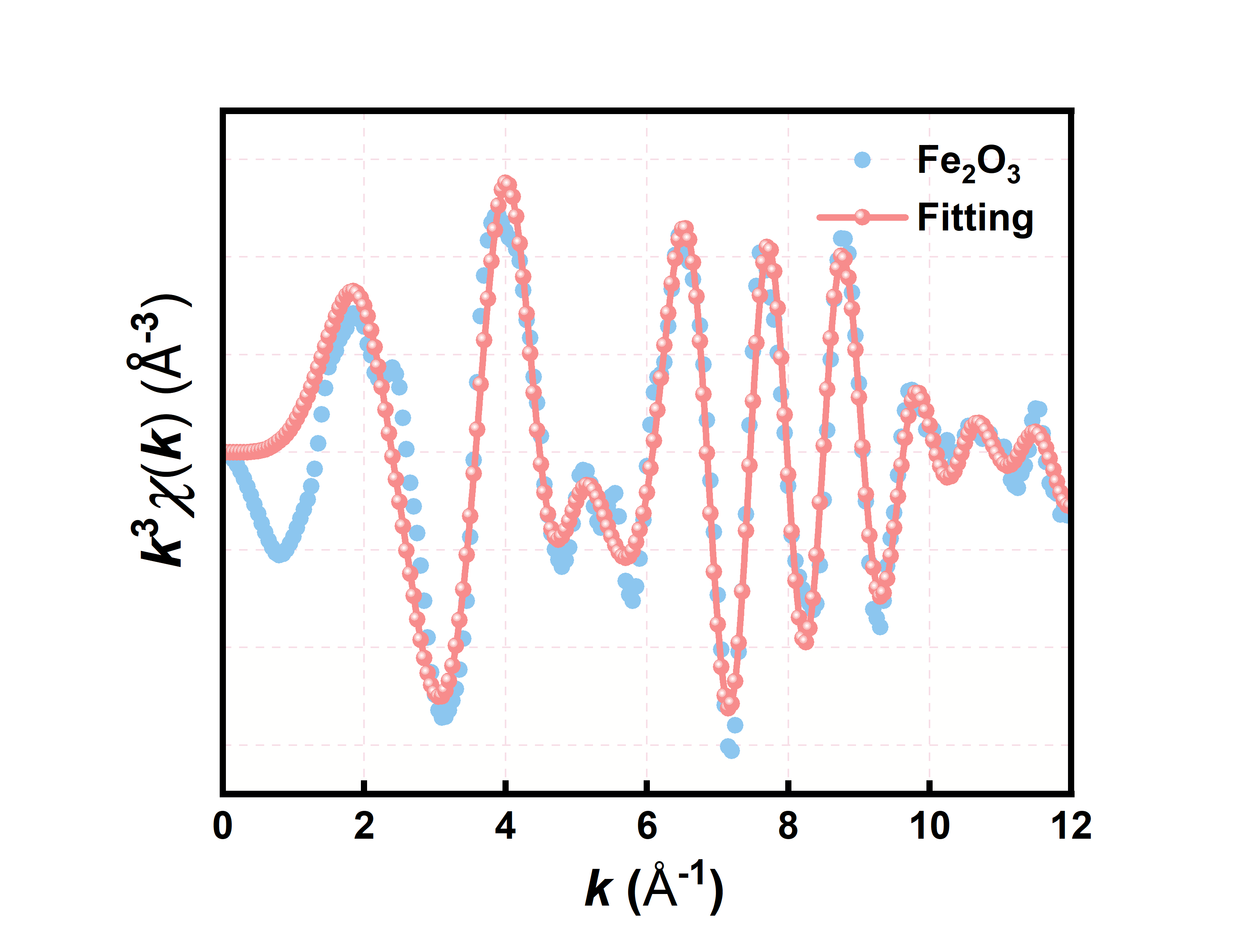


**Figure S8**. EXAFS fitting curves of Fe_2_O_3_ at the k space.


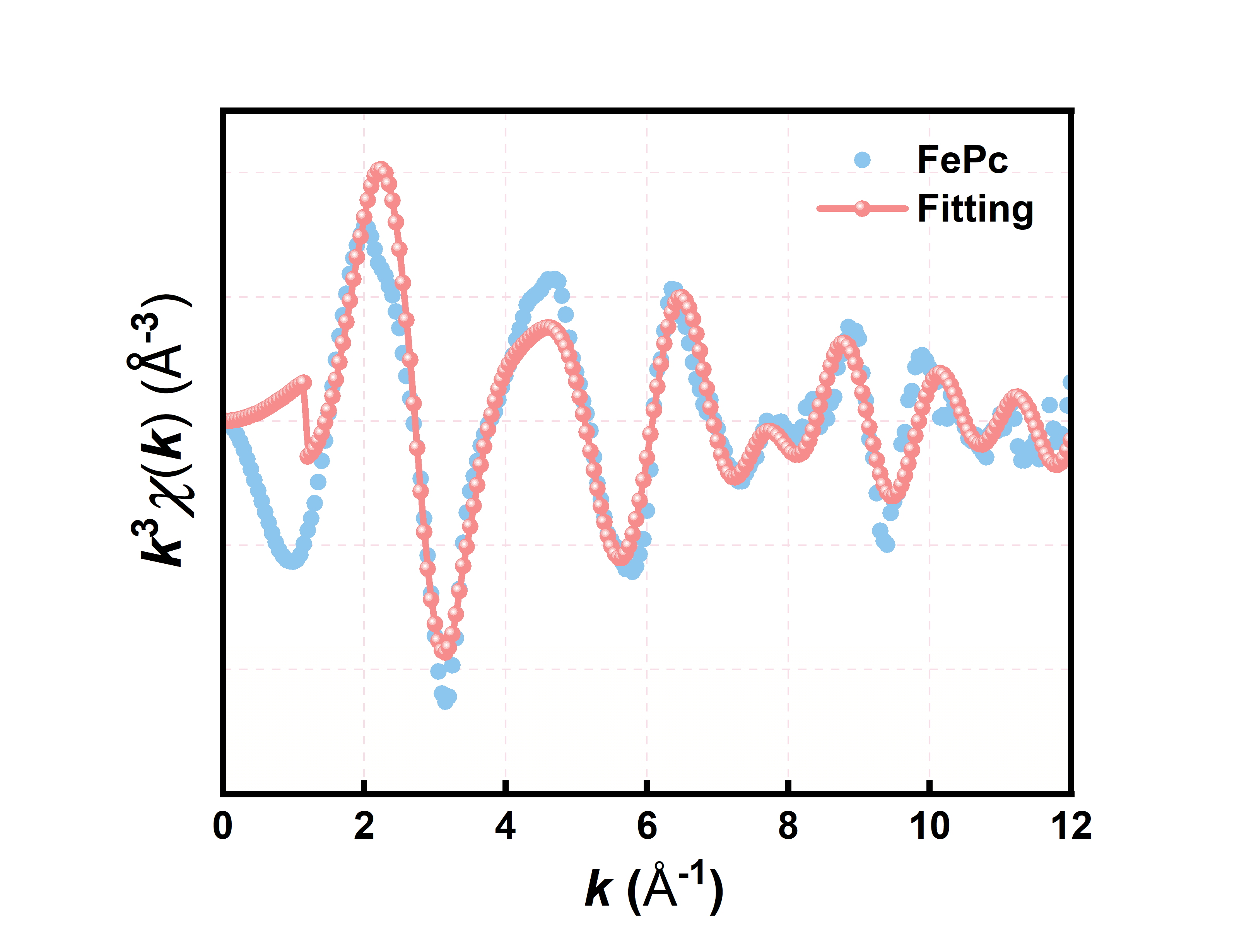


**Figure S9**. EXAFS fitting curves of FePc at the k space.

**Table S1.** EXAFS data fitting results of Samples.

| Sample | Path | *CN^a^* | *R*(Å)*^b^* | *σ*^2^ (Å^2^)*^c^* | Δ*E*_0_(eV)*^d^* | *R* factor |
| --- | --- | --- | --- | --- | --- | --- |
| Fe K-edge (*Ѕ*_0_^2^=0.759) | | | | | | |
| Fe foil | Fe-Fe | 8.0* | 2.469±0.007 | 0.0043 | 5.7 | 0.0025 |
|  | Fe-Fe | 6.0* | 2.854±0.008 | 0.0057 |  |  |
| Fe_2_O_3_ | Fe-O | 3.3±0.2 | 1.932±0.009 | 0.0034 | -2.3 | 0.0049 |
|  | Fe-O | 2.3±0.2 | 2.095±0.014 |  |  |  |
|  | Fe-Fe | 5.1±0.2 | 2.955±0.006 | 0.0065 | -1.3 |  |
|  | Fe-Fe | 4.0±0.3 | 3.382±0.008 |  |  |  |
|  | Fe-Fe | 5.3±0.4 | 3.689±0.006 |  |  |  |
| FePc | Fe-N | 3.9±0.3 | 1.971±0.009 | 0.0064 | 4.9 | 0.0193 |
|  | Fe-N-C | 5.3±0.7 | 2.977±0.015 | 0.0030 |  |  |
| Fe/SAE | Fe-N | 3.9±0.3 | 2.006±0.023 | 0.0031 | 4.3 | 0.0081 |


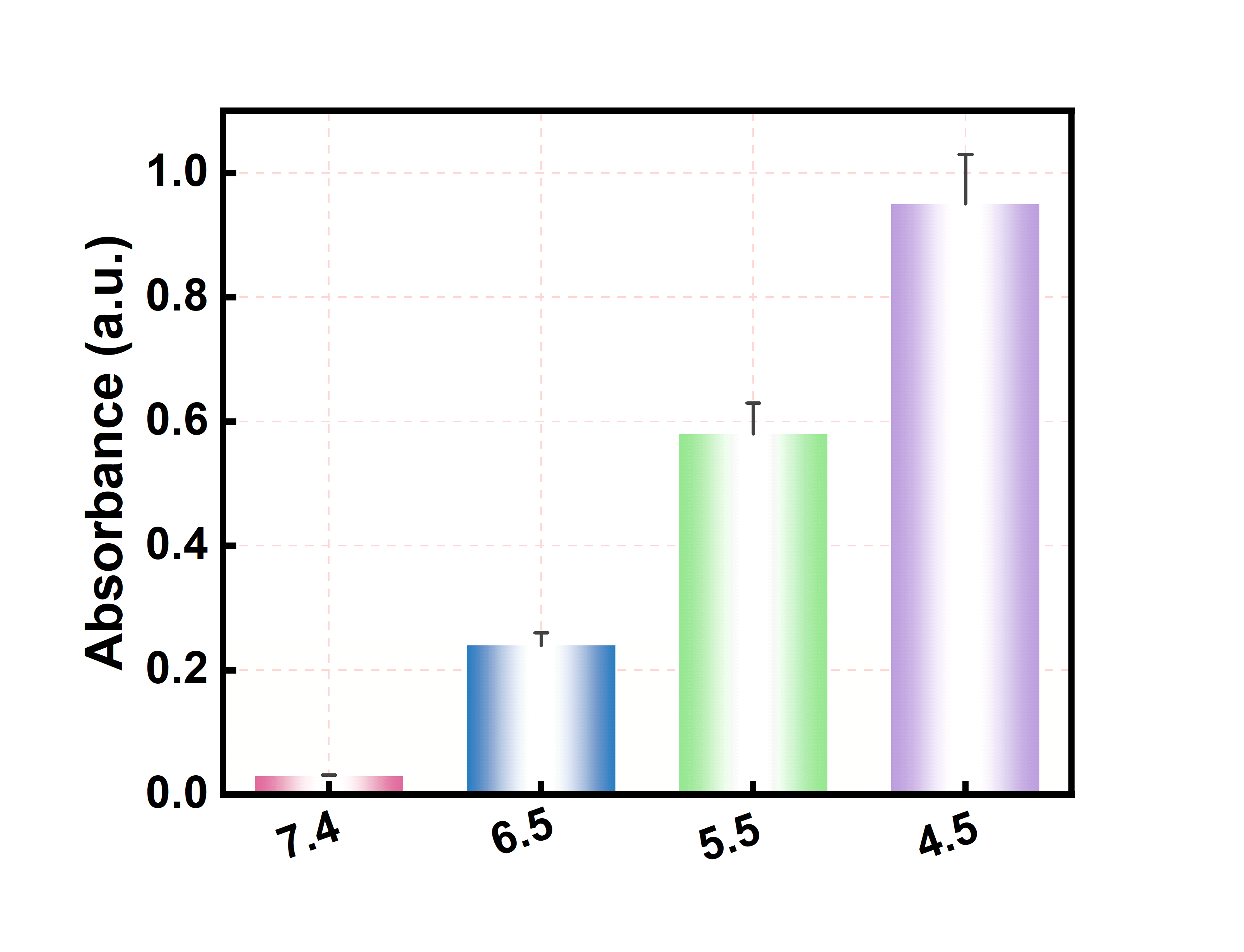


**Figure S10**. TMB assay for measuring POD-like activity of the Fe/SAE at the different pH.


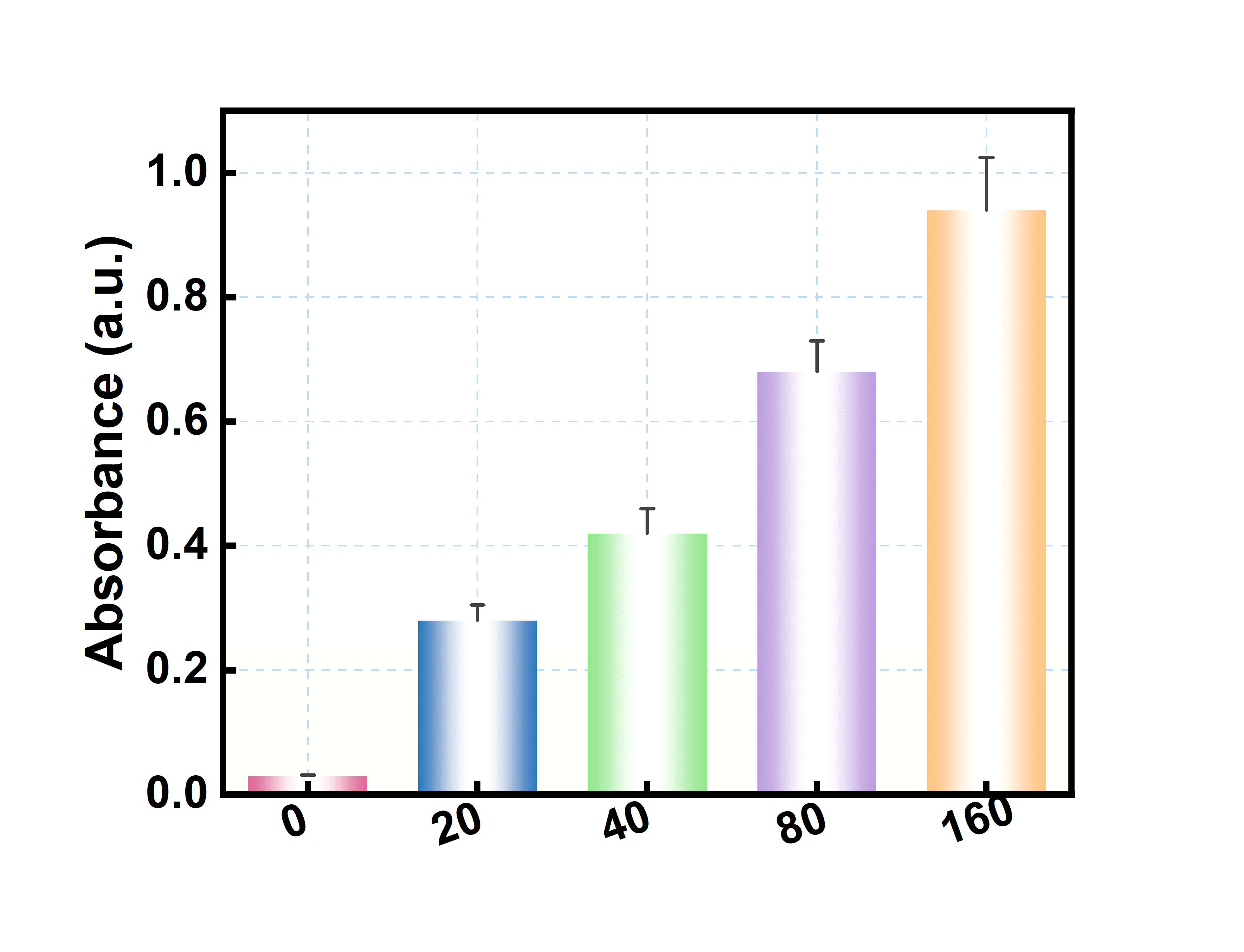


**Figure S11**. TMB assay for measuring POD-like activity after incubating the different concentrations of Fe/SAE.


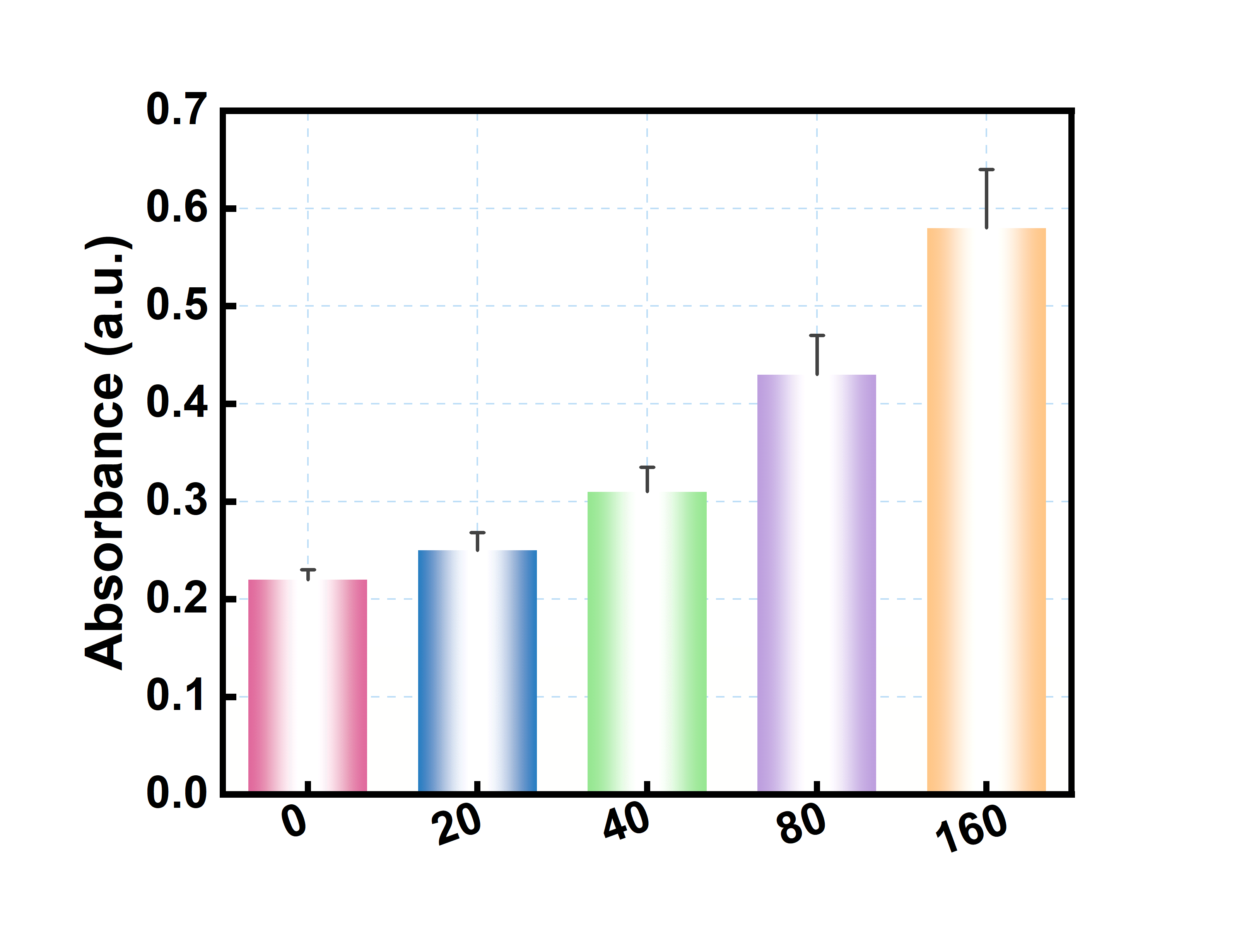


**Figure S12**. ABTS assay for measuring POD-like activity after incubating the different concentrations of Fe/SAE.


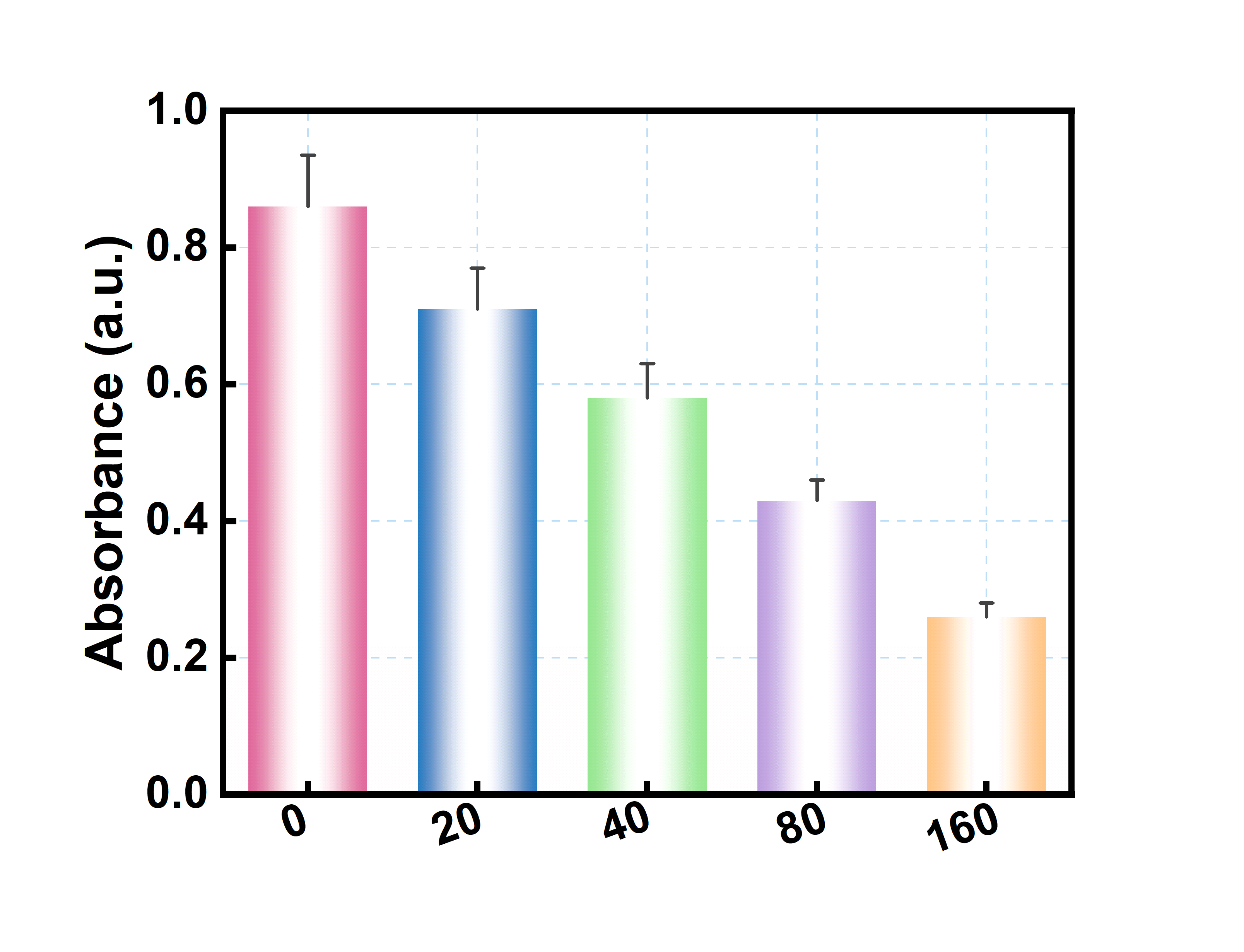


**Figure S13**. MB assay for measuring POD-like activity after incubating the different concentrations of Fe/SAE.


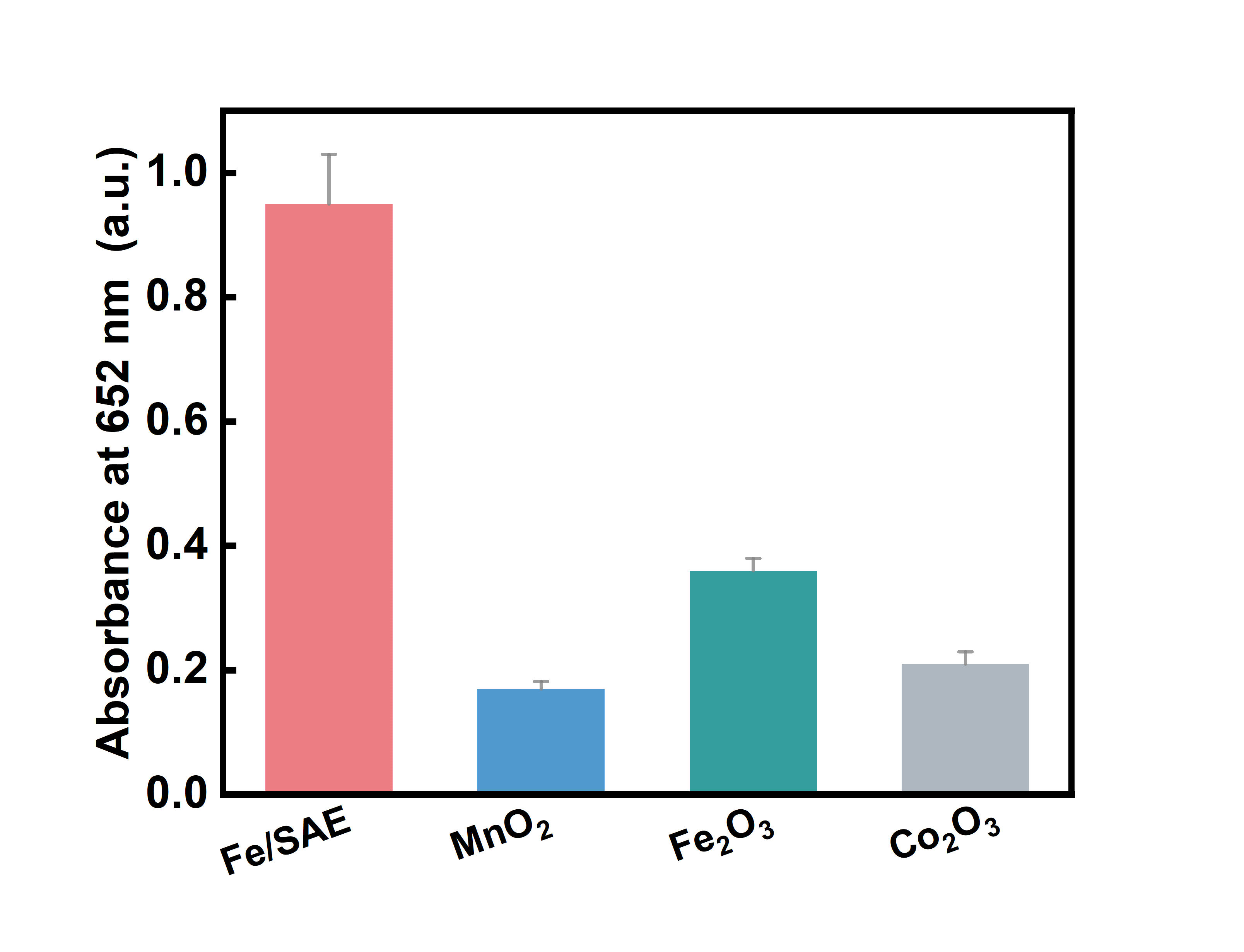


**Figure S14.** The POD-like activity of different nanozymes based on TMB assay.


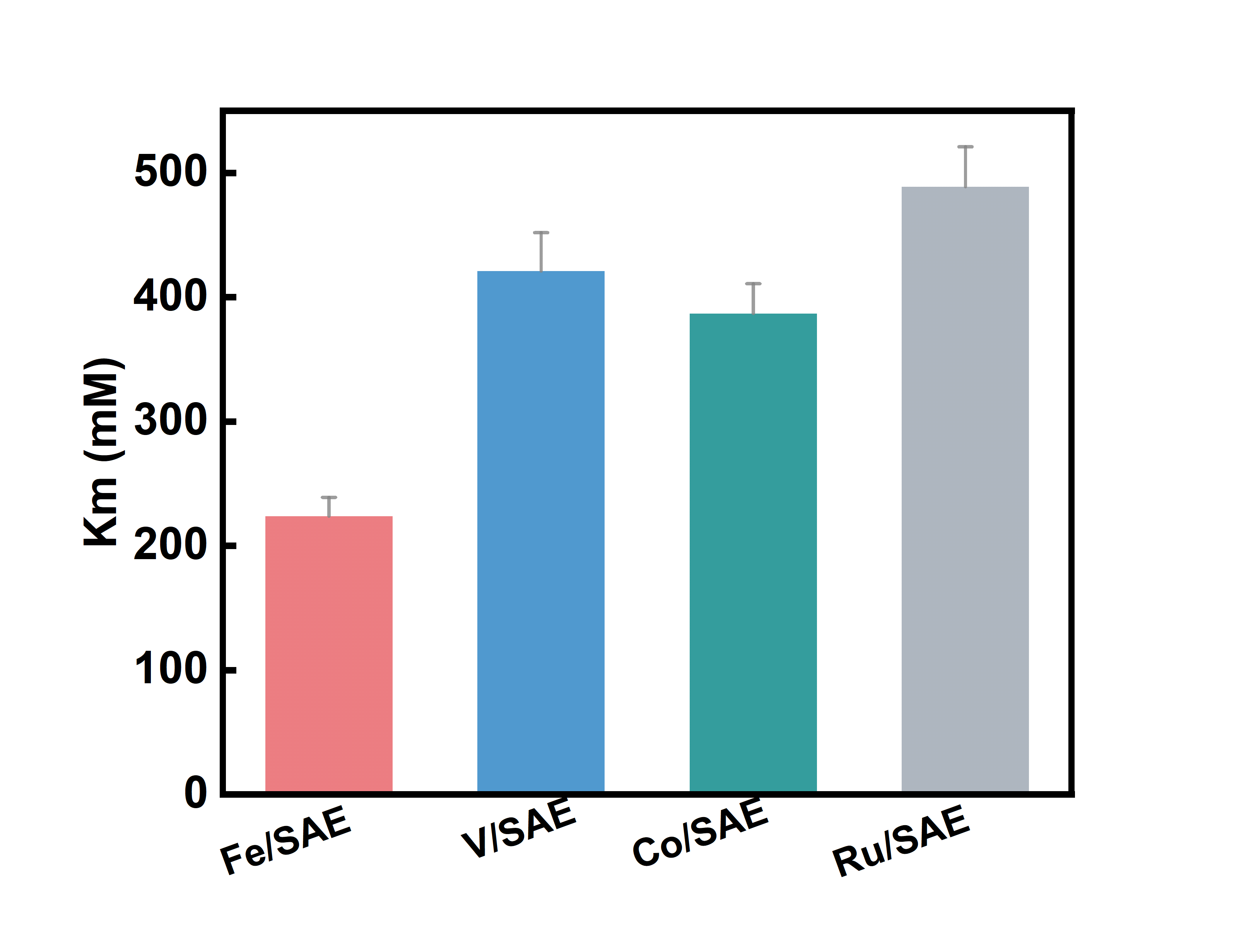


**Figure S15.** The Km values of different single atom nanozymes.


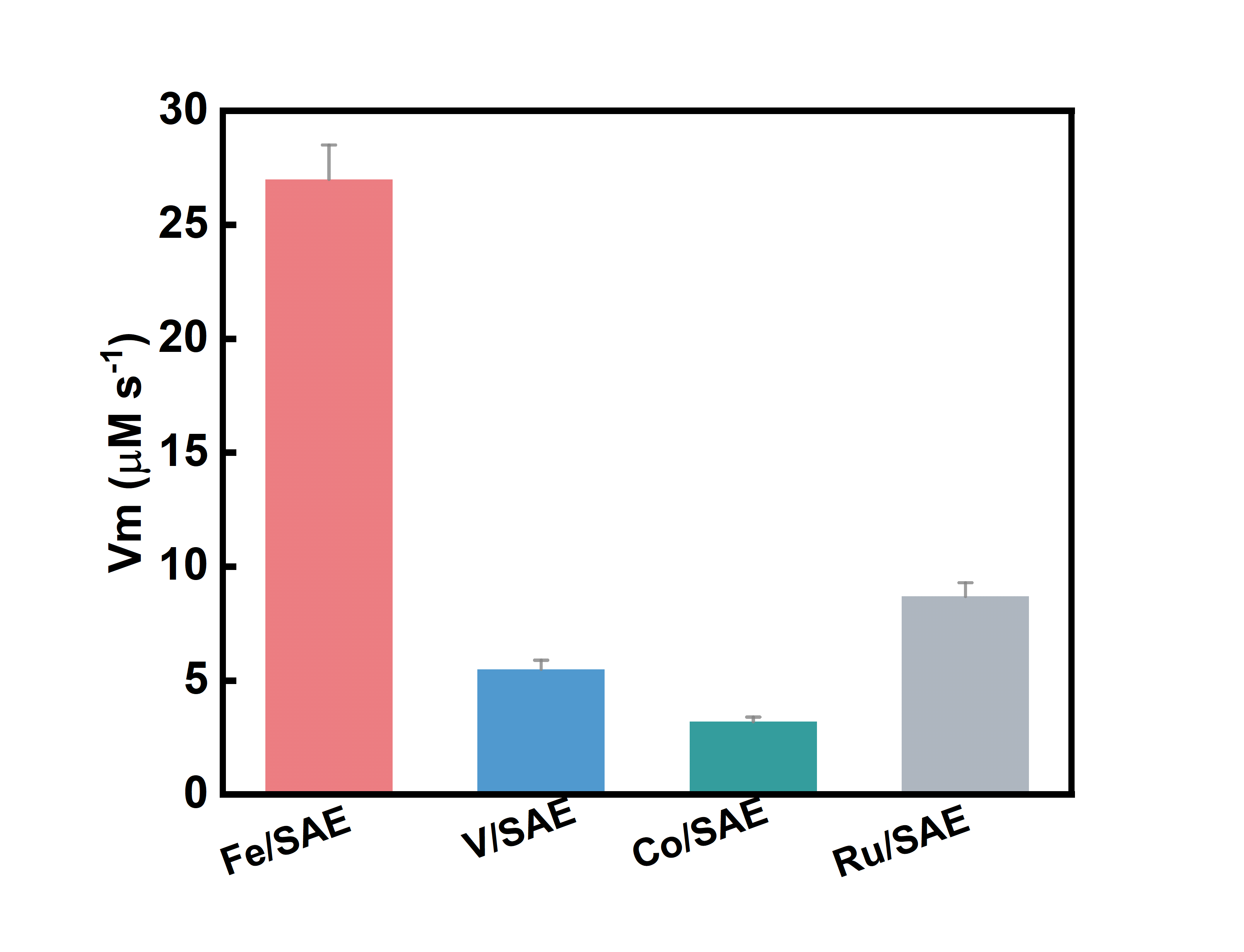


**Figure S16.** The Km values of different single atom nanozymes.


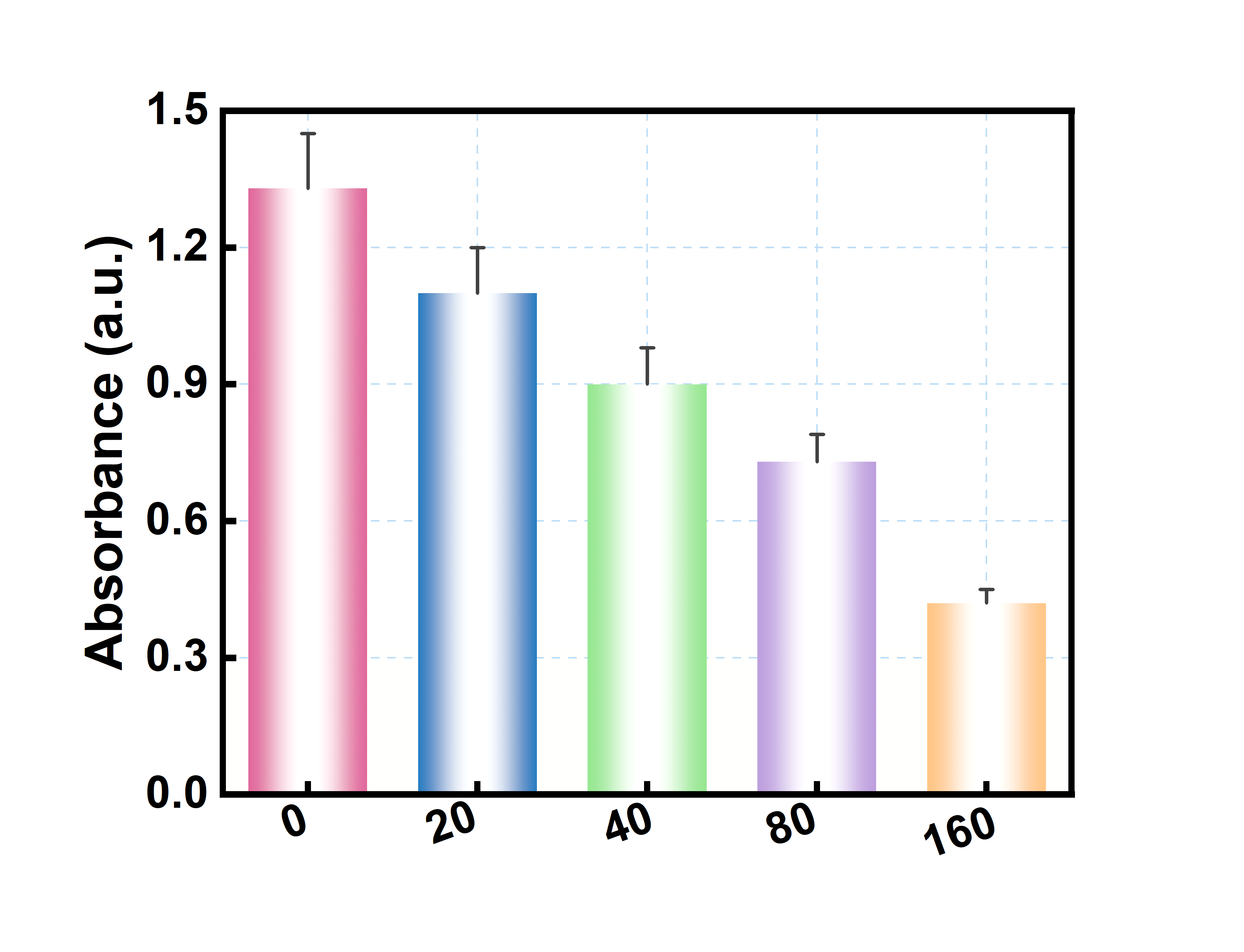


**Figure S17**. ABTS assay for measuring GSHOX-like activity after incubating the different concentrations of Fe/SAE.


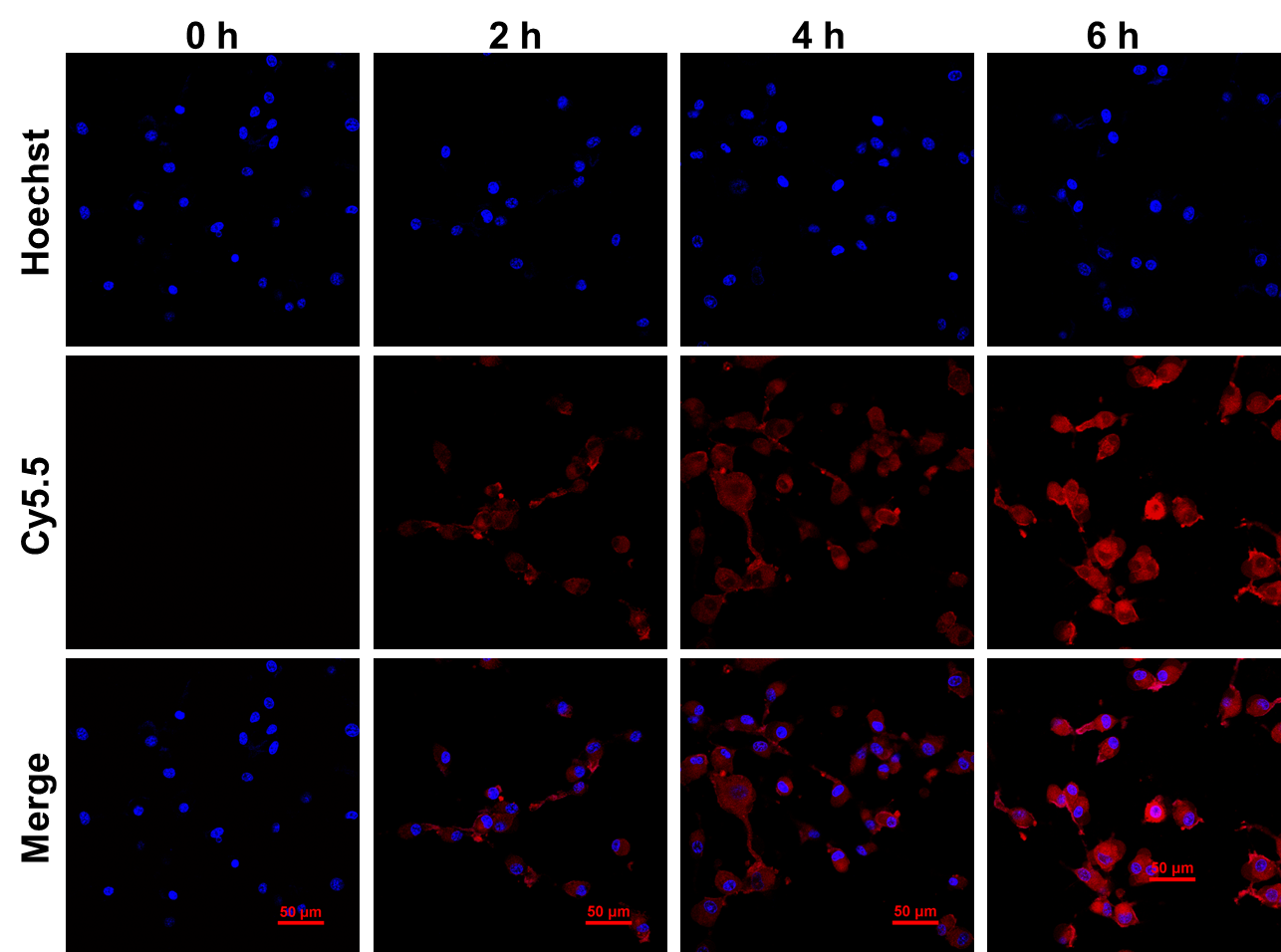


**Figure S18**. The CLSM images of tumor cells incubated with Cy5.5-labeled Fe/SAE@A.


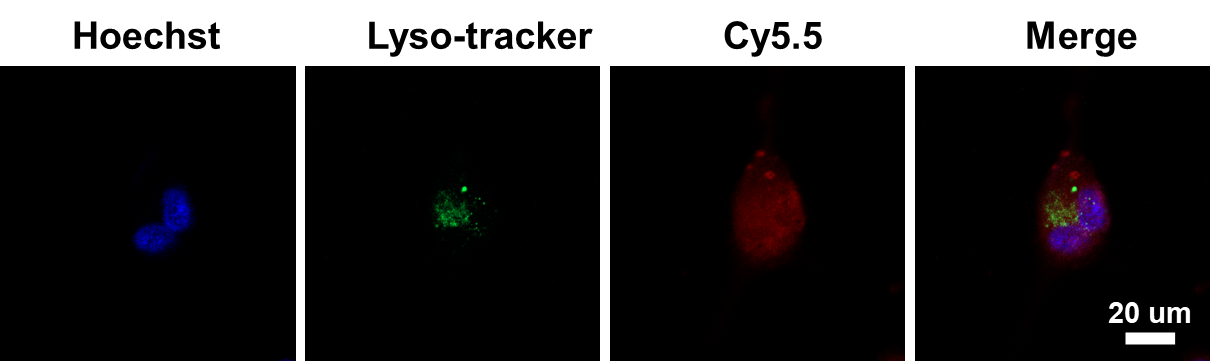


**Figure S19**. The CLSM images of GL261 cells colocalization.


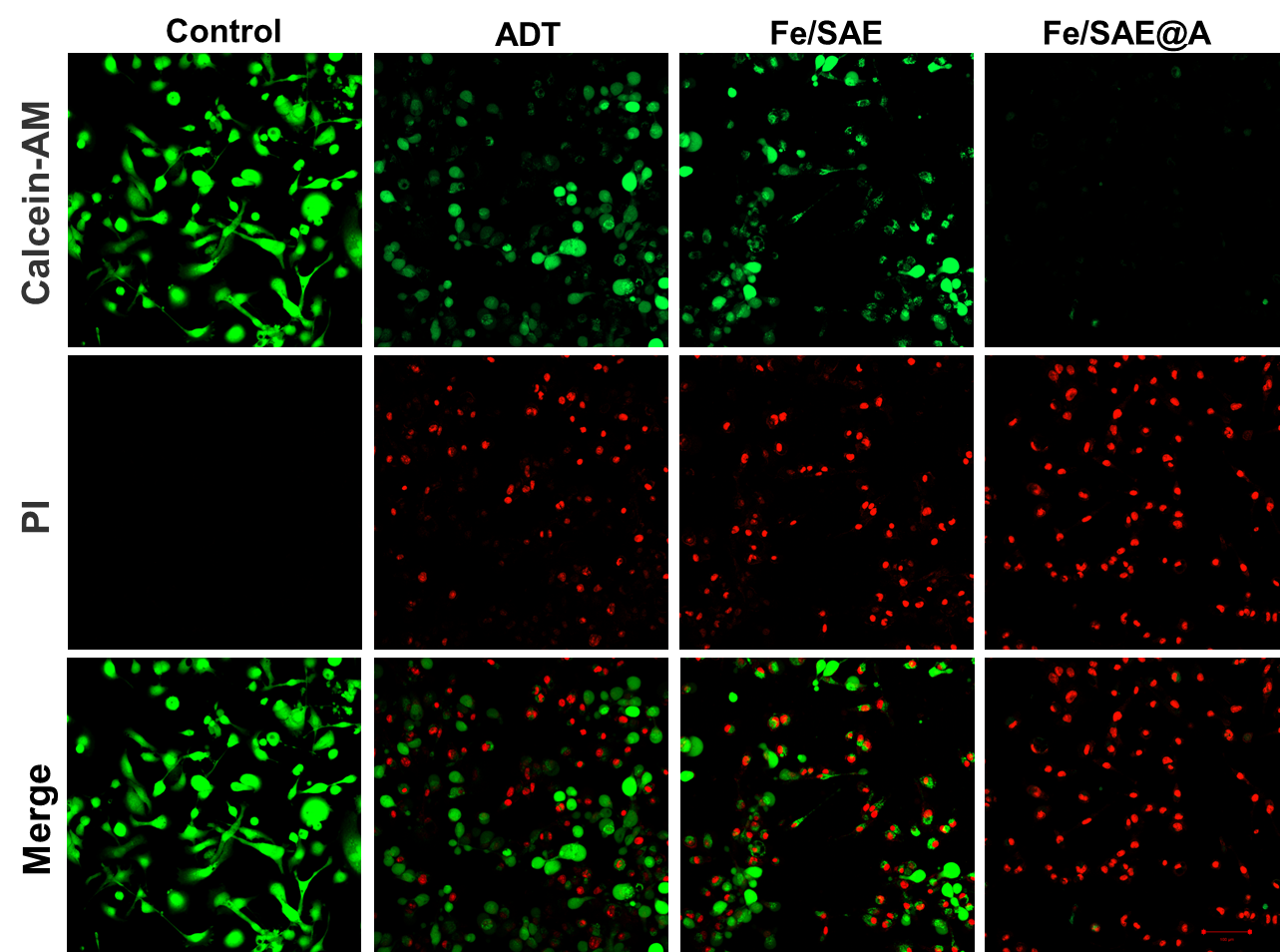


**Figure S20**. Calcein-AM/PI co-stained GL261 cells incubated with various formulations.


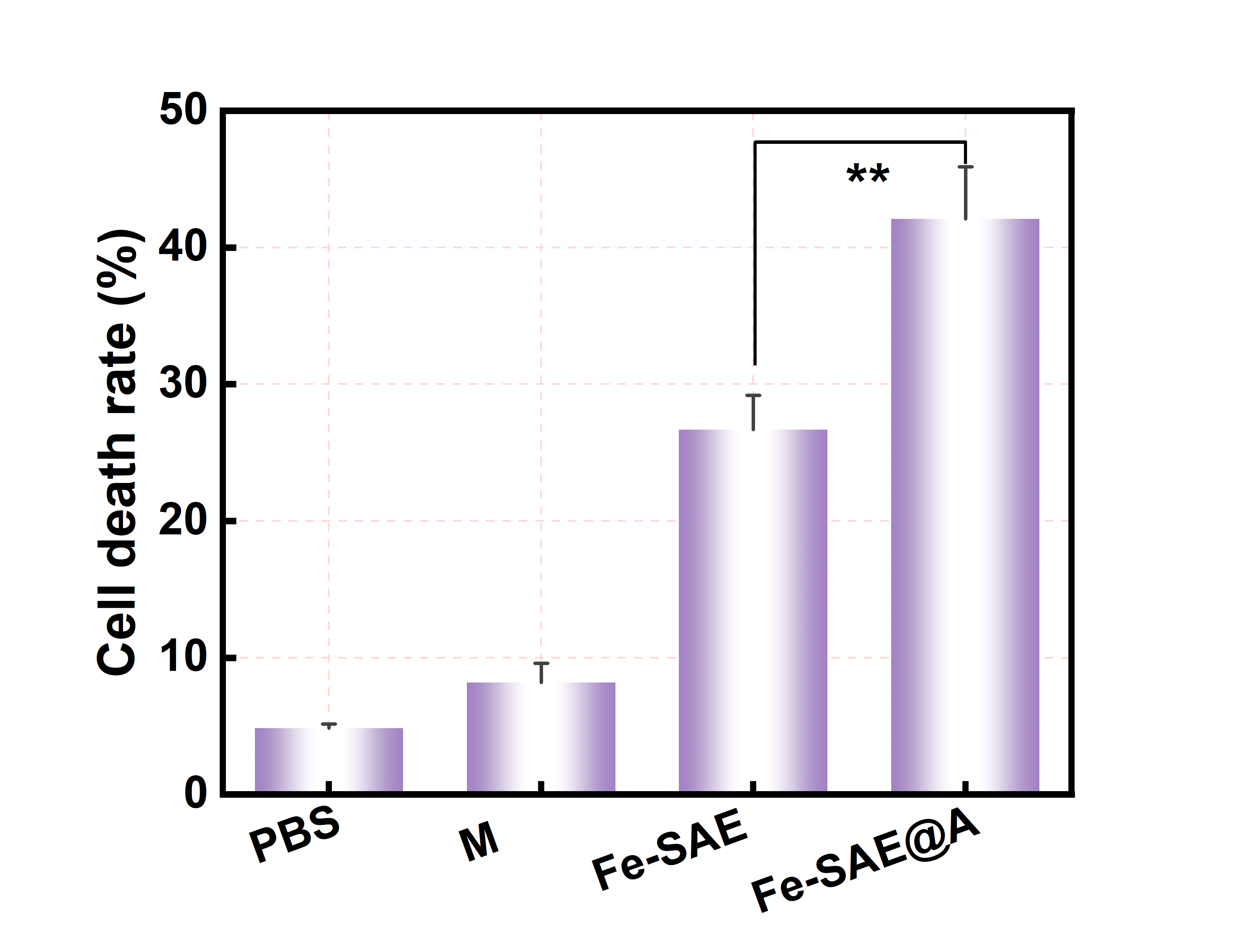


**Figure S21**. Flow cytometry measurements of GL261 cells after incubation with varying formulations.


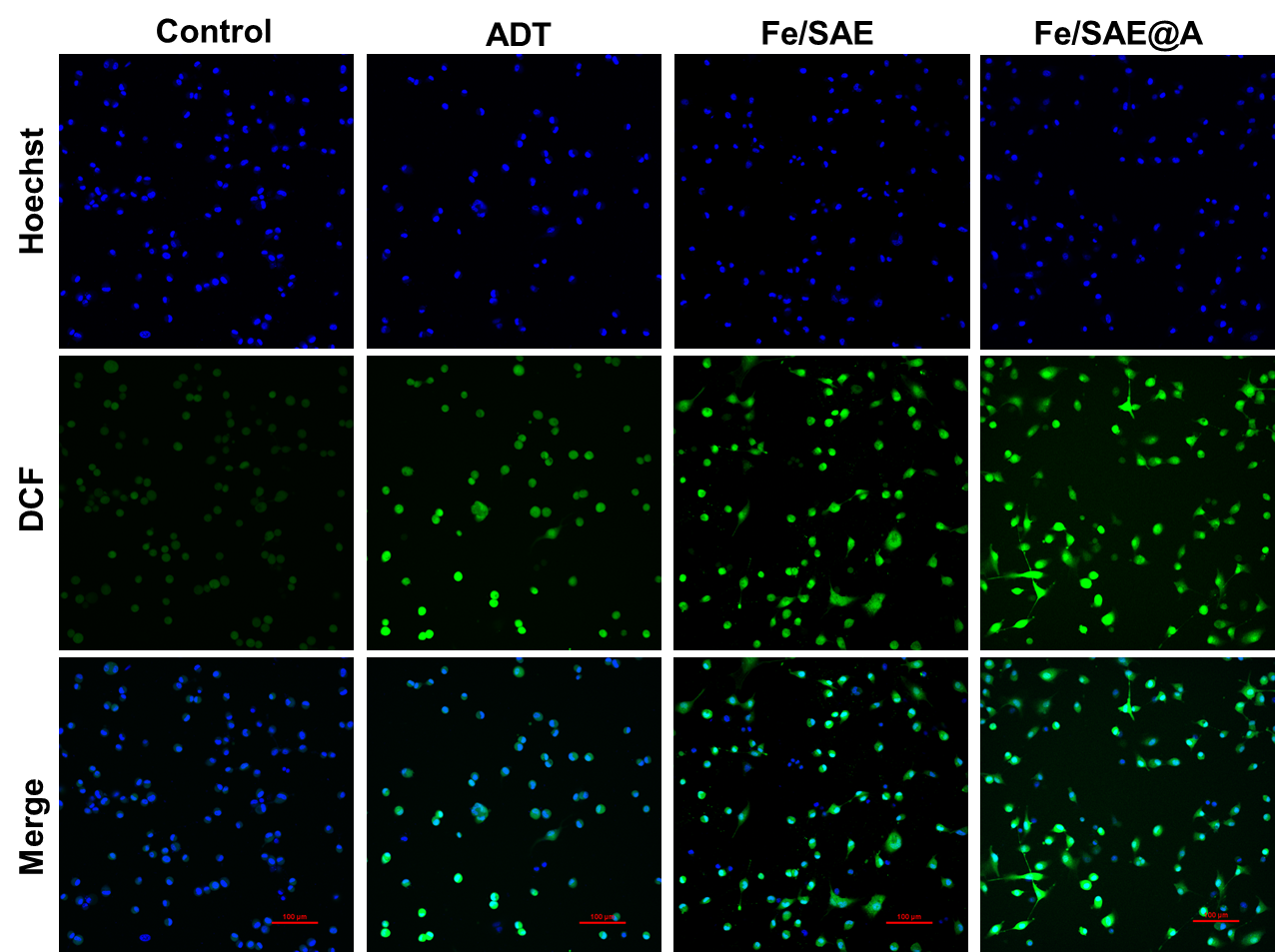


**Figure S22**. CLSM images of DCF in GL261 cells treated with different formulations.


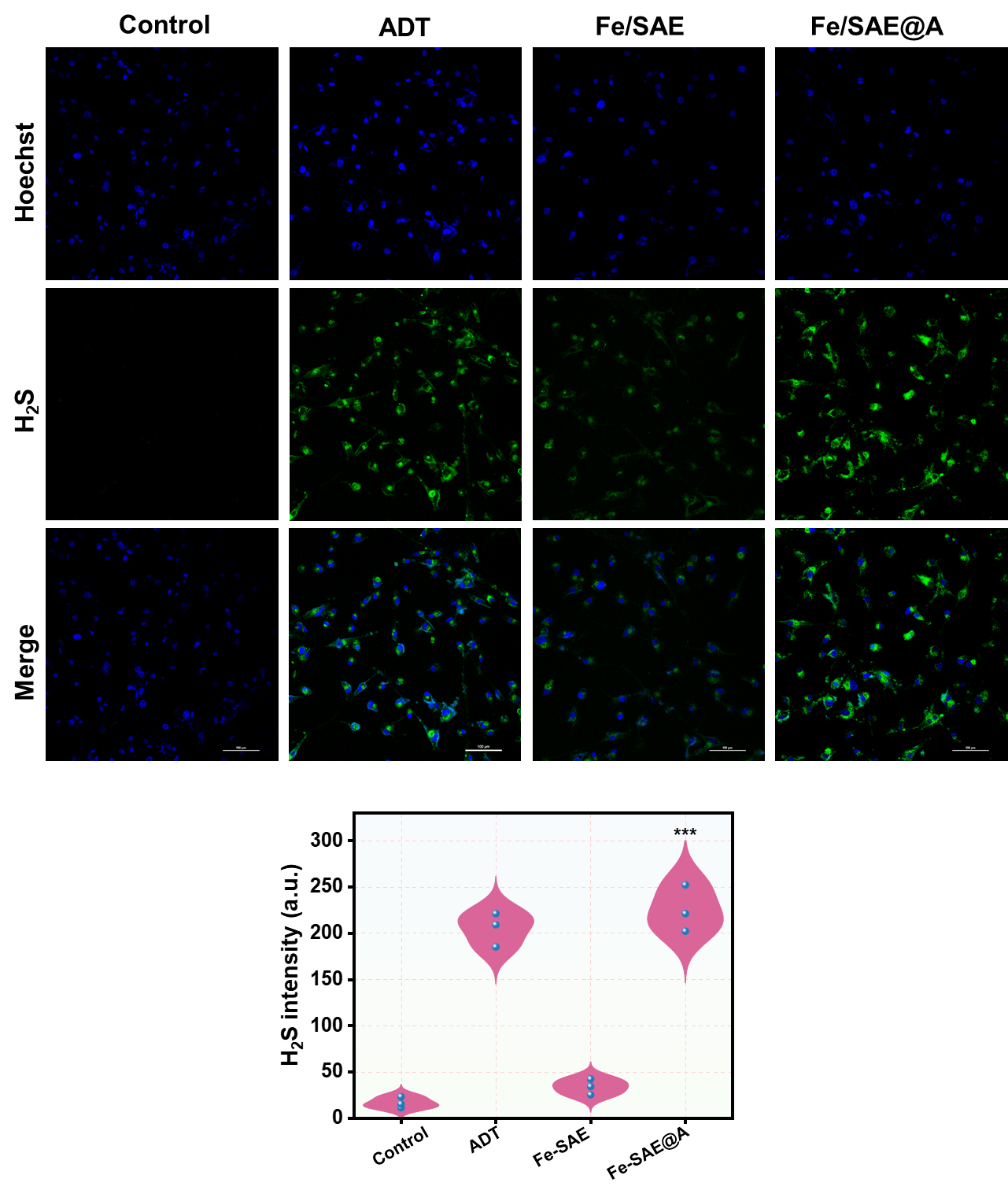


**Figure S23**. CLSM images of H_2_S in GL261 cells treated with different formulations.


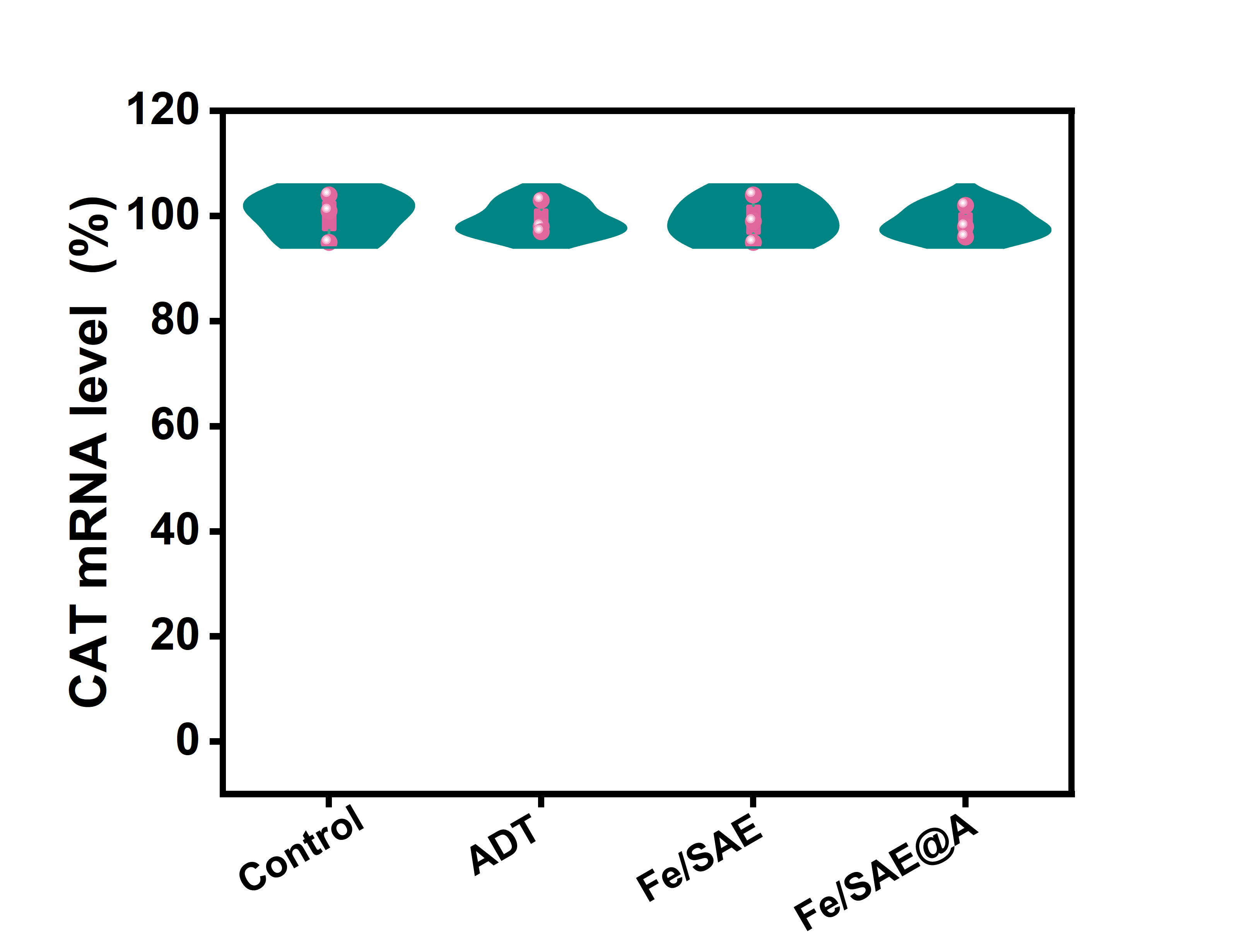

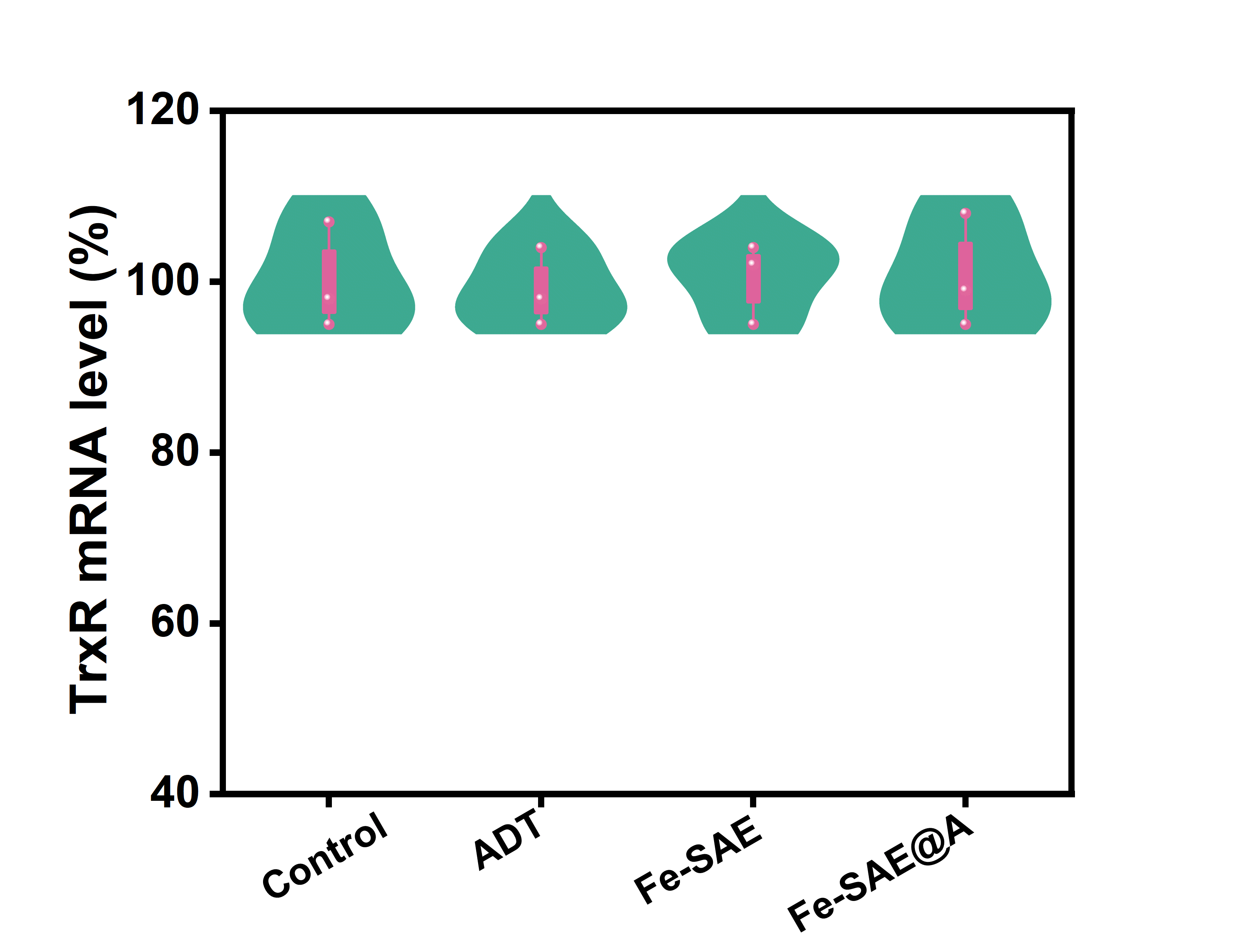


**Figure S24.** qRT-PCR of CAT and TrxR mRNA levels.


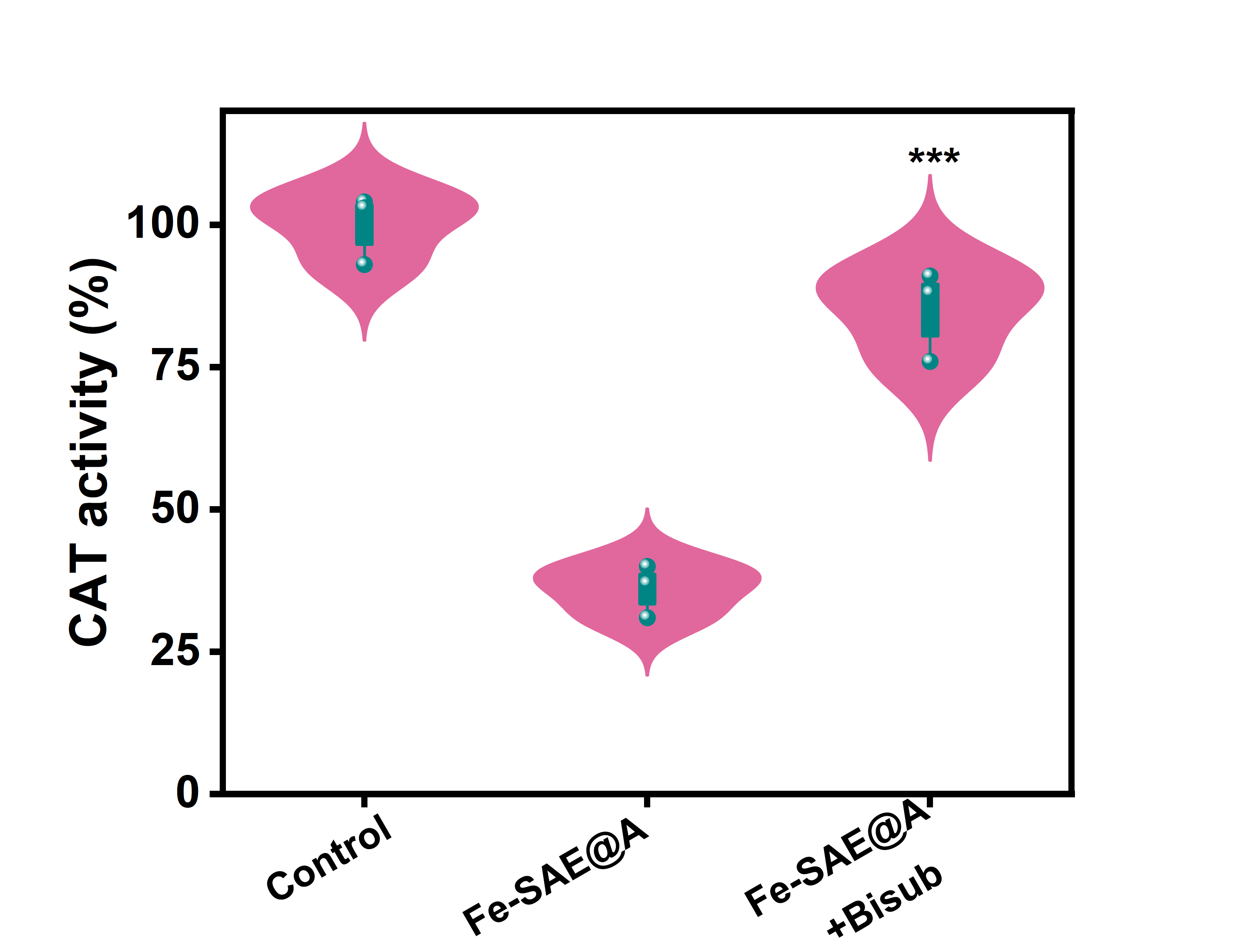

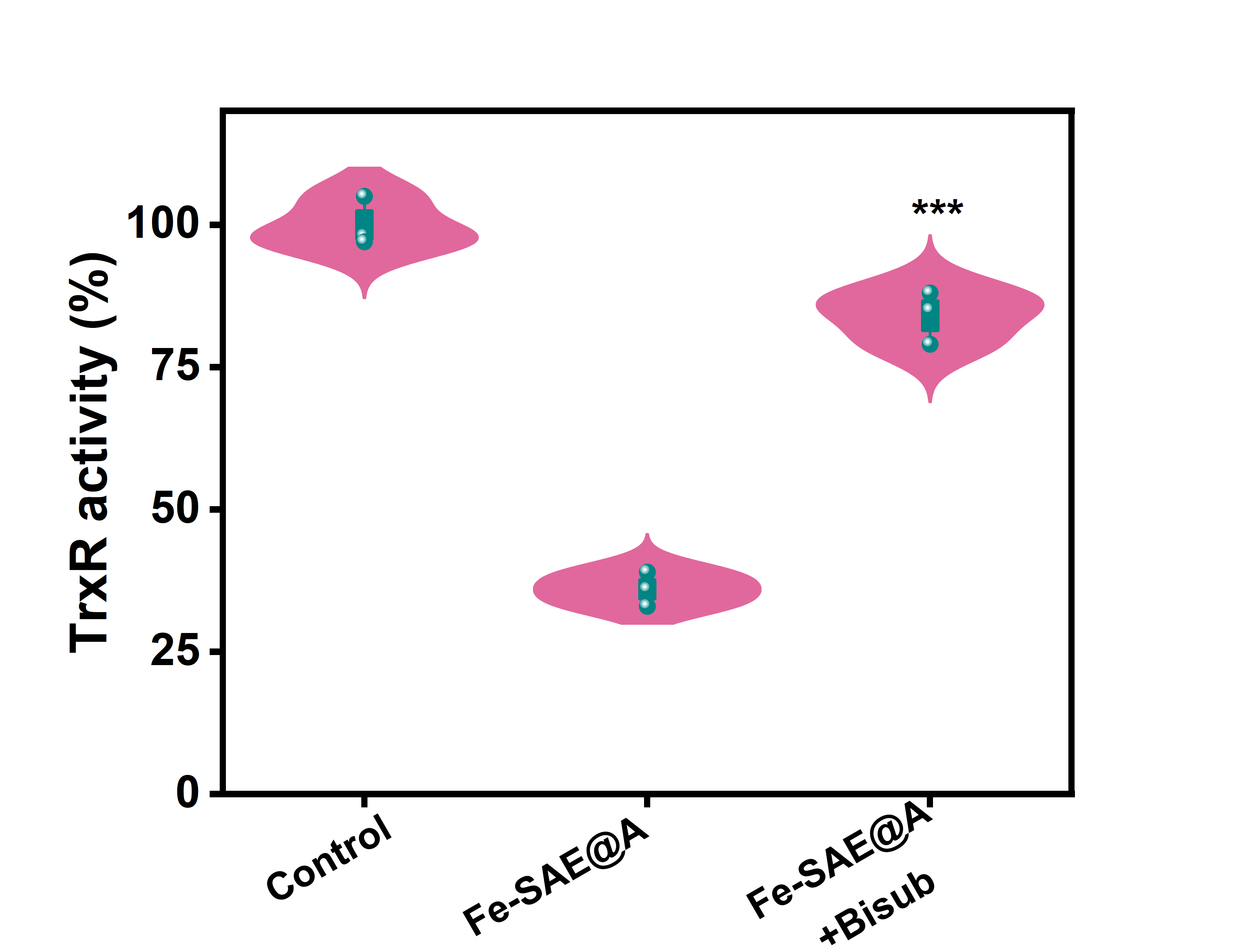


**Figure S25.** CAT and TrxR activity following different treatments.


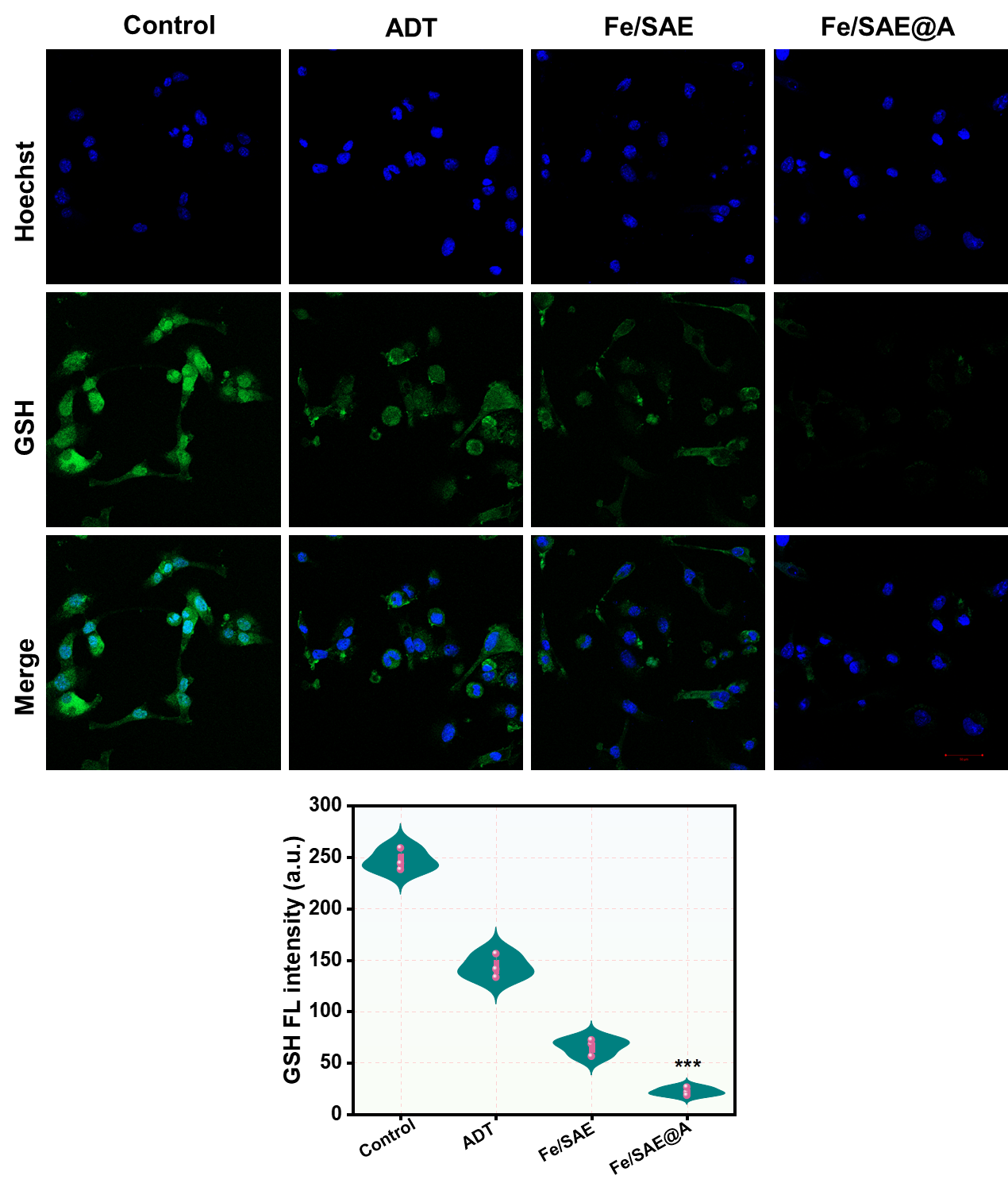


**Figure S26**. CLSM images of GSH in GL261 cells treated with different formulations.


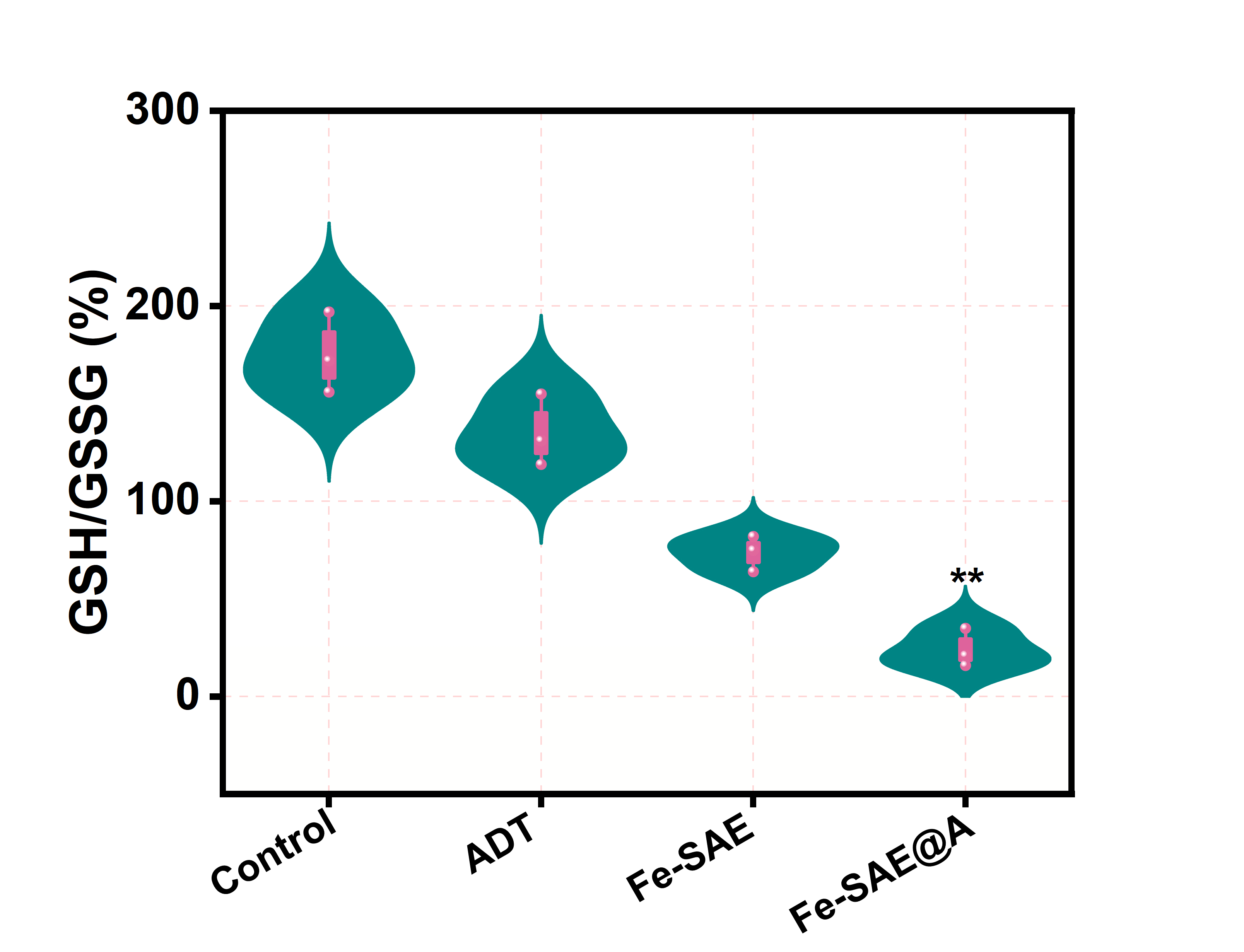


**Figure S27**. GSH/GSSG level in GL261 cells after different formulations.


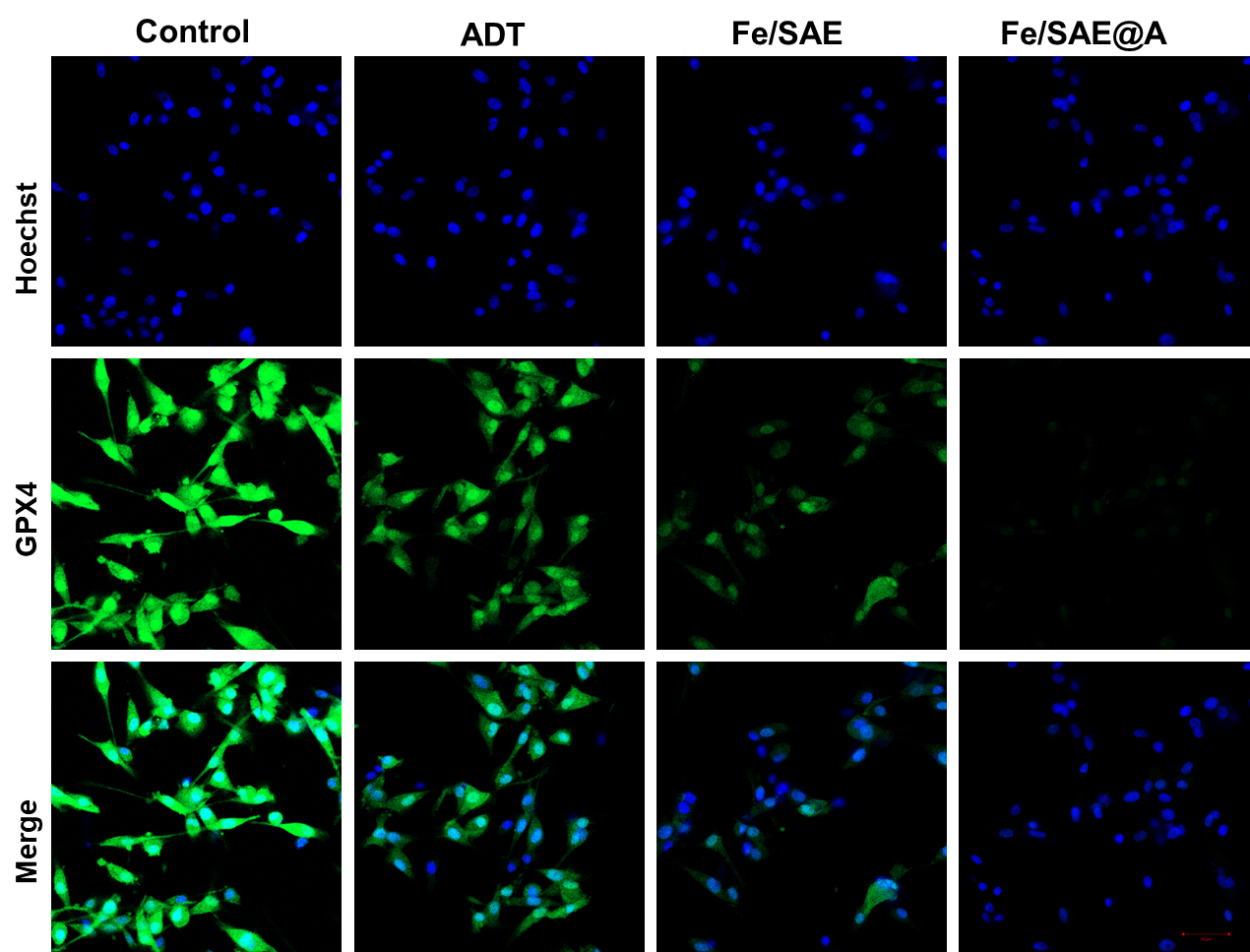


**Figure S28**. CLSM images of GPX4 expression in GL261 cells treated with different formulations.


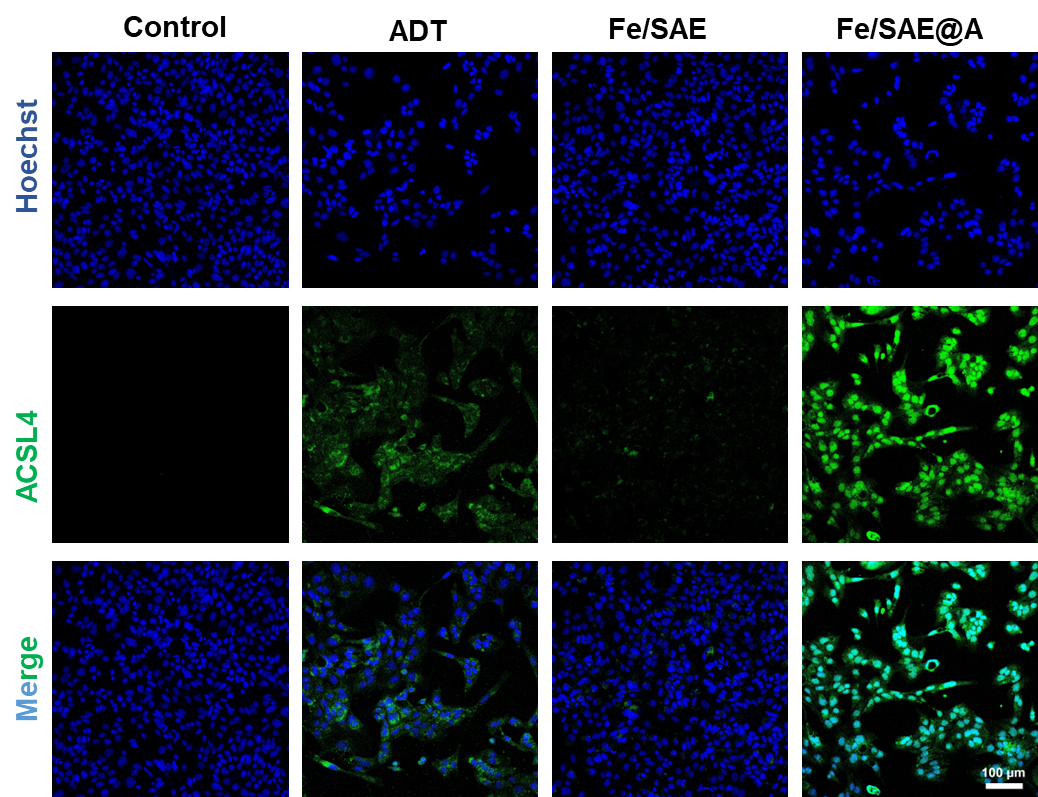


**Figure S29.** The ACSL4 expression within tumor cells following different treatments.


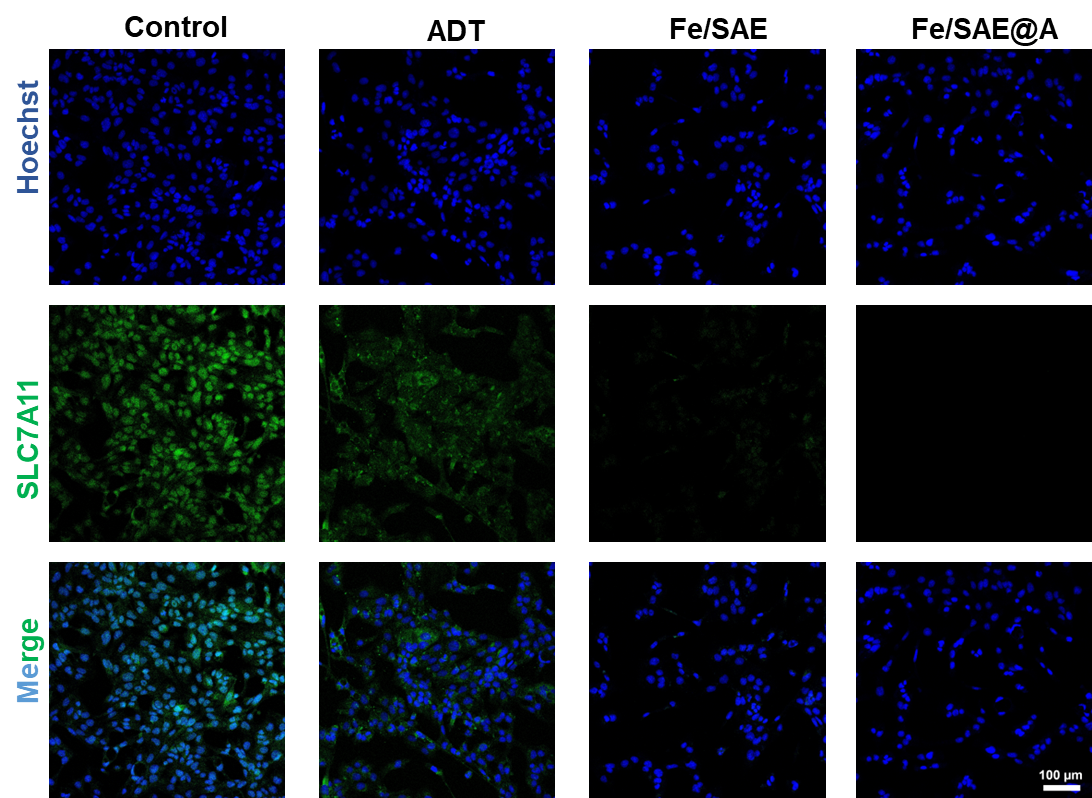


**Figure S30.** The SLC7A11 expression within tumor cells following different treatments.


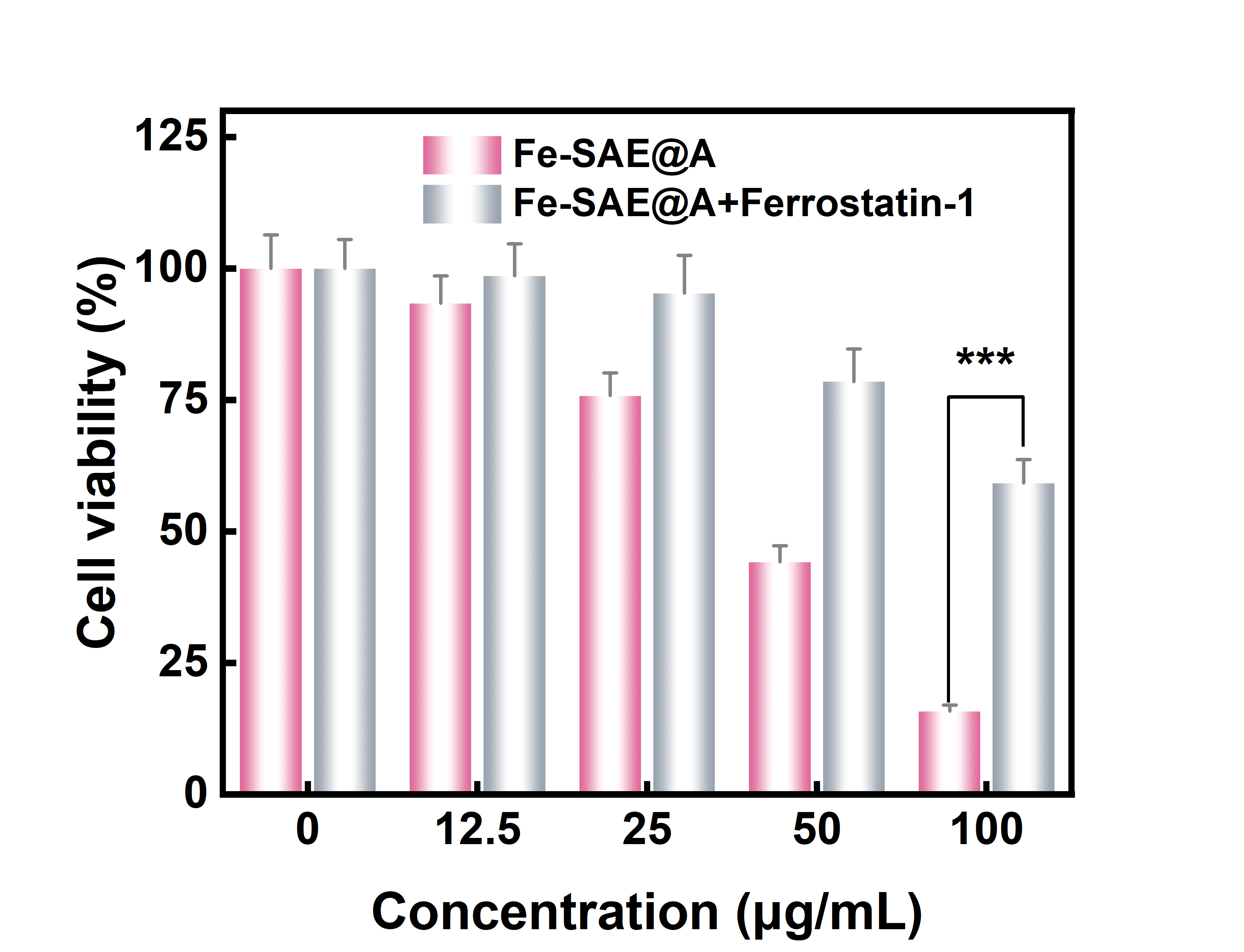


**Figure S31.** The cell death rate was measure using CCK-8 kit.


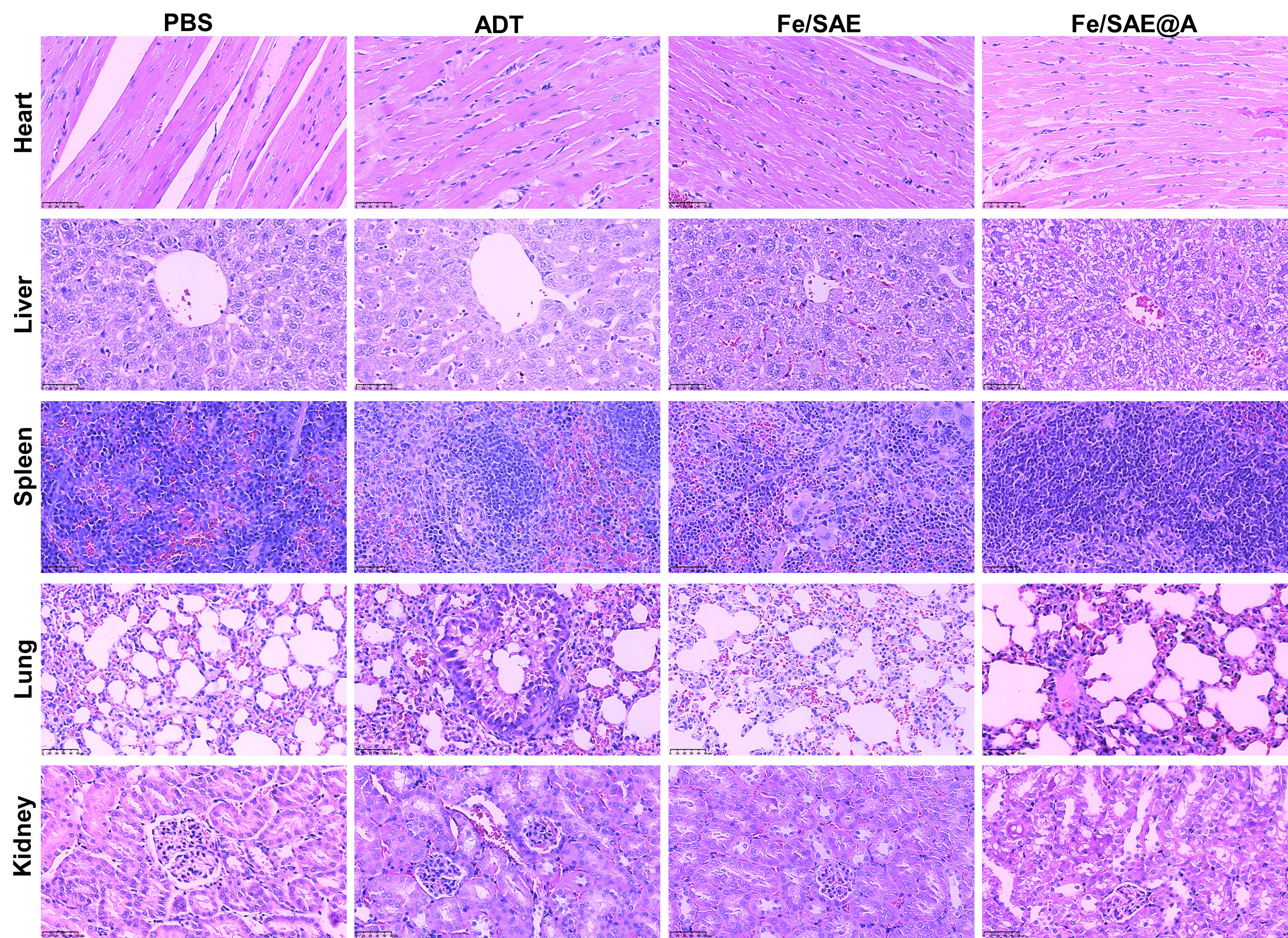


**Figure S32**. H&E staining of the major organs.


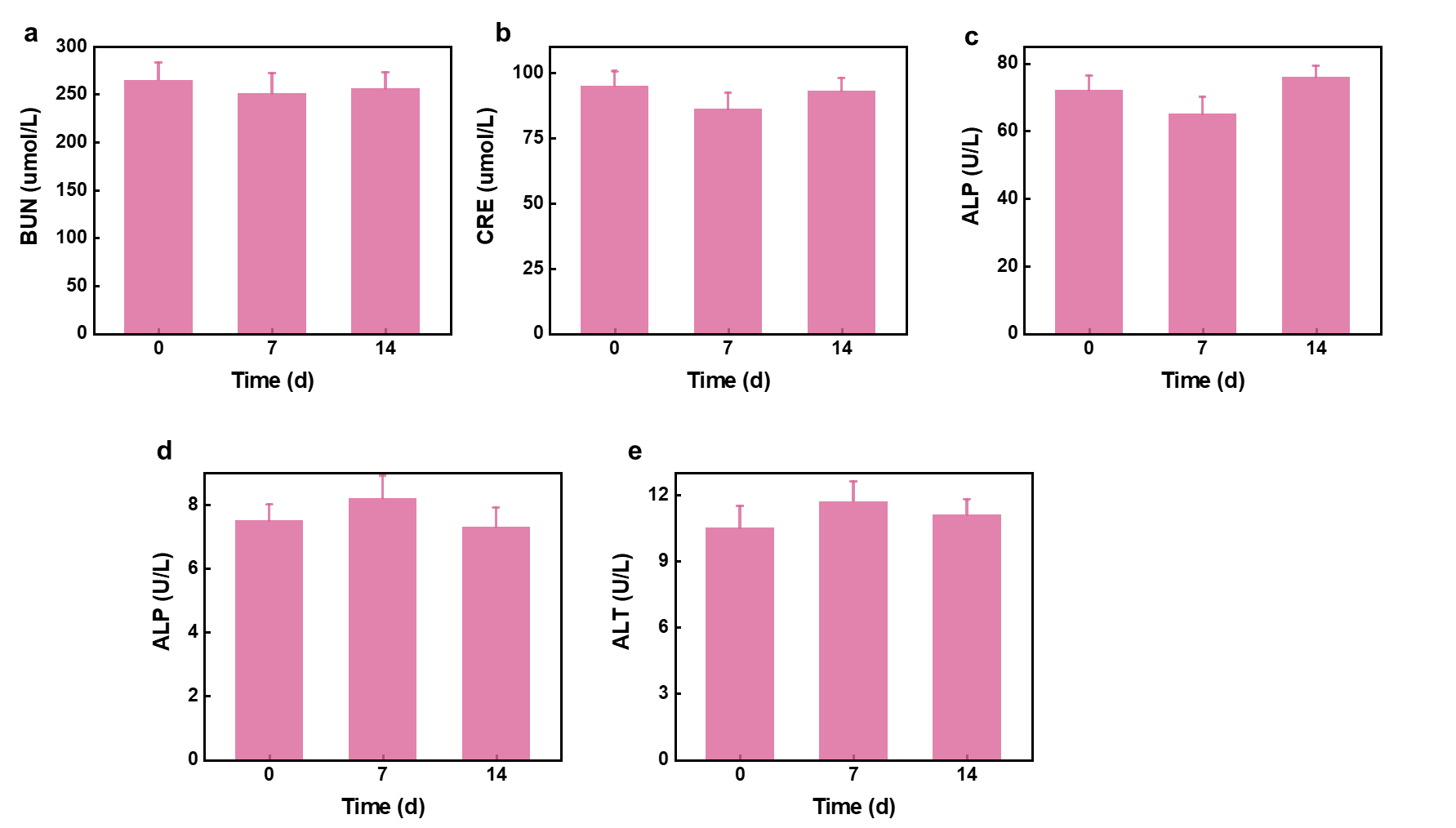


**Figure S33.** The change of biochemical factors following Fe/SAE@A injection.


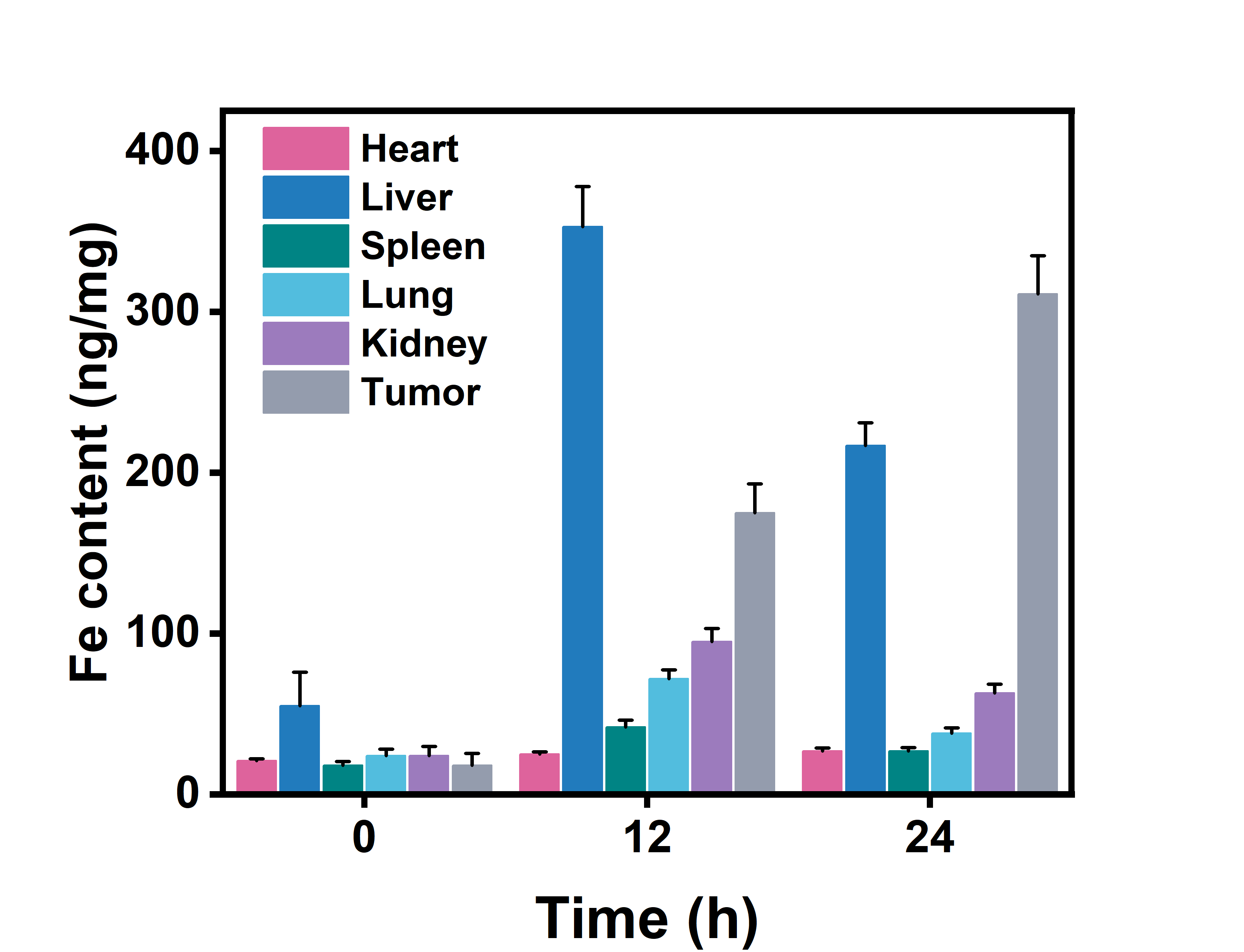


**Figure S34.** The biodistribution of Fe/SAE@A.


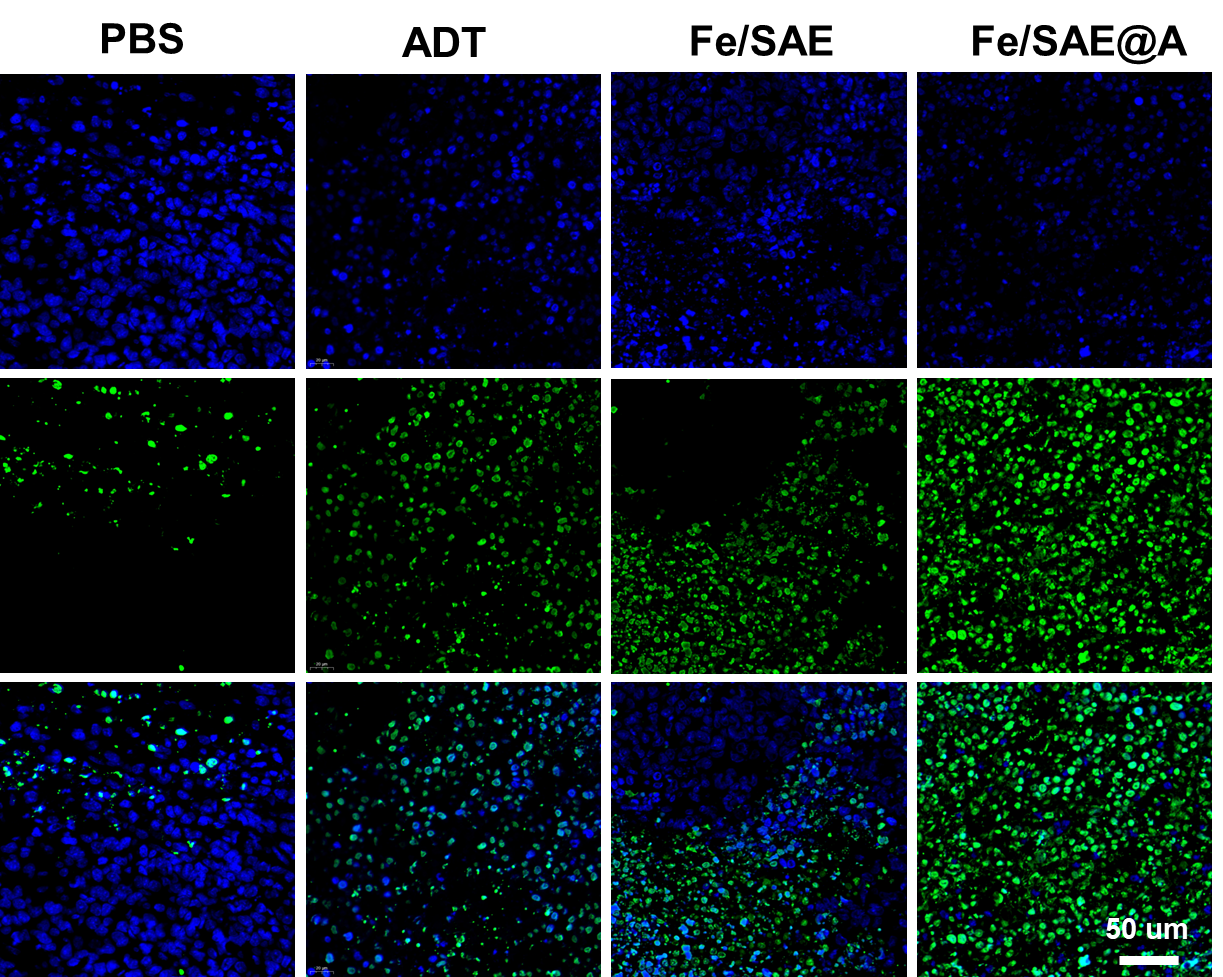


**Figure S35.** TUNEL staining of tumor tissue.


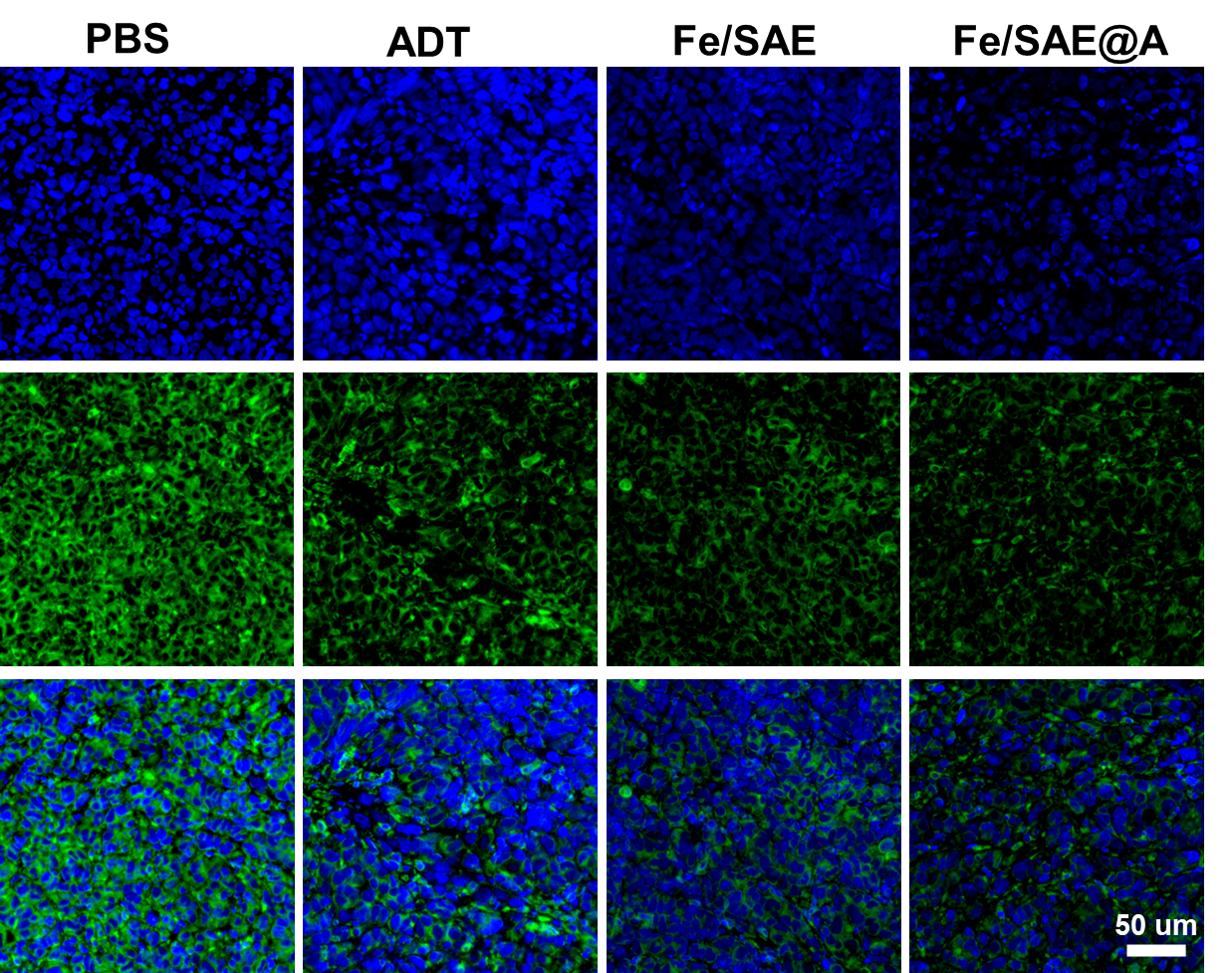


**Figure S36.** GPX4 staining of tumor tissue.


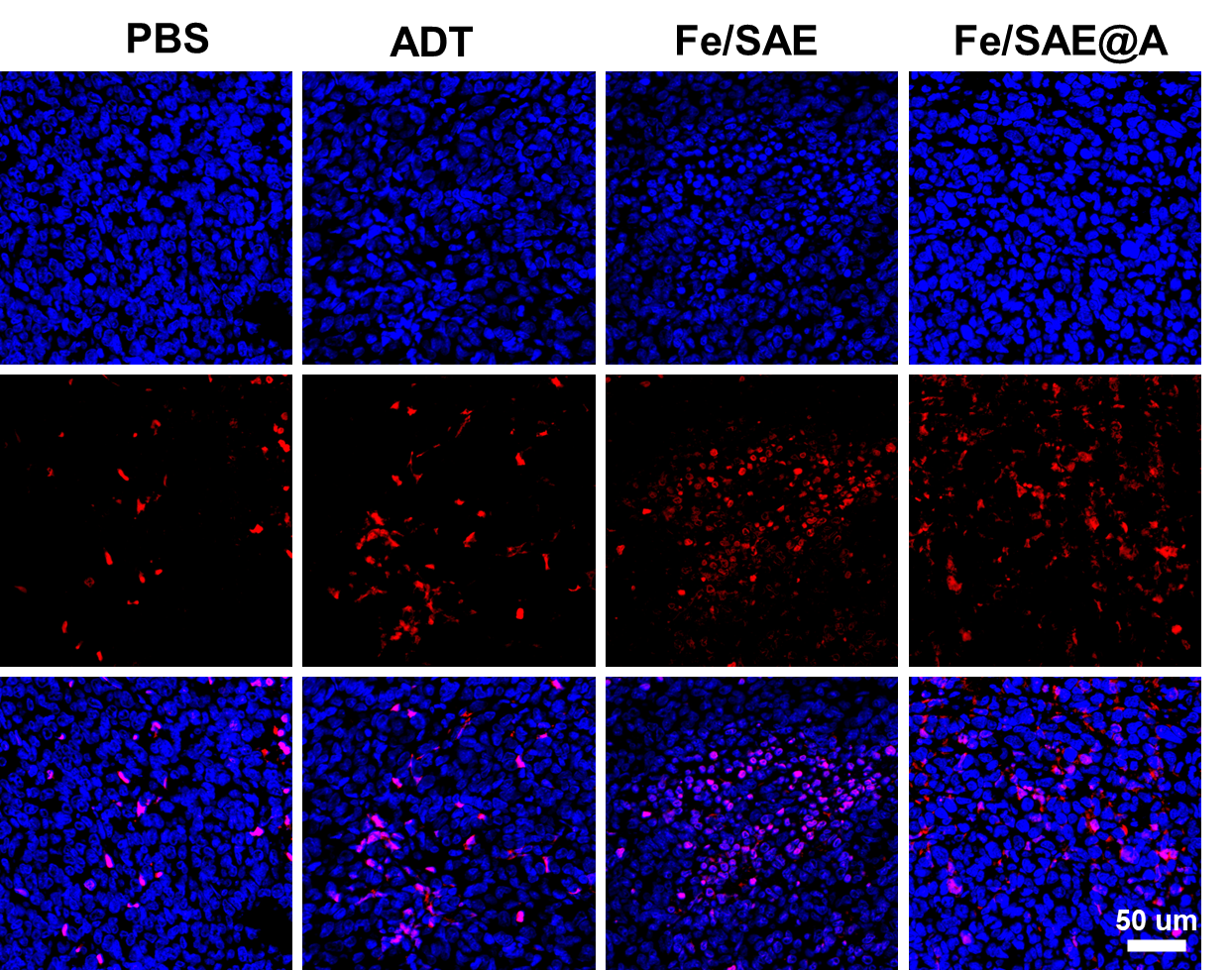


**Figure S37.** ROS staining of tumor tissue.
